# The generalized Wiener–Hopf equations for the elastic wave motion in angular regions

**DOI:** 10.1098/rspa.2021.0624

**Published:** 2022-01

**Authors:** Vito G. Daniele, Guido Lombardi

**Affiliations:** DET-Poltecnico di Torino, 10129 Torino, Italy

**Keywords:** wave motion, wedge, Wiener–Hopf method, integral equations, spectral domain, elasticity

## Abstract

In this work, we introduce a general method to deduce spectral functional equations in elasticity and thus, the generalized Wiener–Hopf equations (GWHEs), for the wave motion in angular regions filled by arbitrary linear homogeneous media and illuminated by sources localized at infinity. The work extends the methodology used in electromagnetic applications and proposes for the first time a complete theory to get the GWHEs in elasticity. In particular, we introduce a vector differential equation of first-order characterized by a matrix that depends on the medium filling the angular region. The functional equations are easily obtained by a projection of the reciprocal vectors of this matrix on the elastic field present on the faces of the angular region. The application of the boundary conditions to the functional equations yields GWHEs for practical problems. This paper extends and applies the general theory to the challenging canonical problem of elastic scattering in angular regions.

## 1. Introduction

In [1], we applied a general theory to obtain spectral functional equations in electromagnetics and thus generalized Wiener–Hopf Equations (GWHEs) for scattering problems in angular regions filled by arbitrarily linear media, inspired by [2] and described also in [3]. The monographs [4,5] show the efficacy of the generalization of the Wiener–Hopf (WH) technique in practical electromagnetic wave scattering problems in the presence of geometries containing angular regions and/or stratified planar regions, see references therein.

In this paper, we implement for the first time the methodology to the challenging canonical problem of elastic scattering in angular regions where some physical quantities are tensors. The technique consists of three steps: (1) the deduction of functional equations in spectral domain of sub-regions that constitute the whole geometry of the problem, (2) the imposition of boundary conditions to get the GWHEs and (3) the solution of the system of the WH equations using exact or semianalytical approximate techniques of factorization as the Fredholm factorization technique [6,7].

This paper is focused on the first fundamental step and introduces the potentialities to develop the other two steps through validations. We follow the method to obtain the WH equations in spectral domain proposed by Jones [8,9], with the application of Fourier/Laplace transforms directly to the PDE formulation of the problem avoiding the tricky derivation of Green’s function in the natural domain. In this work, we use a first-order differential vector formulation for continuous components of the fields, inspired by Bresler & Marcuvitz in [10] for stratified media in electromagnetics. We note that some of theoretical aspects used in electromagnetics (see [1]) are not available in elasticity or are cumbersome to be extended. For this reason, the GWHEs derivation for scattering by angular regions in elasticity is more complicated and challenging, although following the same general theory. Indeed, the authors of this paper have preliminary introduced in [4,5] an abstract formulation for simplified elastic scattering problems concerning the semi-infinite crack and some initial aspects of wedge problems.

In this paper, we first extend the formulation presented in [1] to elastic problems in angular regions using oblique Cartesian coordinates. It yields a matrix differential problem of first order whose unknowns are the field components continuous to the faces of the angular regions. The application of Laplace transform along one face of the angular region and the assumption of problem invariance along the edge profile yield a matrix ordinary differential problem of first order. Following [1] based on [11], we develop a spectral solution before imposing boundary conditions based on the derivation of the dyadic Green’s functions in terms of eigenvectors and eigenvalue of the algebraic matrix operator (of the first-order differential formulation) .

The projection of the solution on reciprocal vectors allows to get a set of functional equations that relate the Laplace transforms of continuous field components along one face of the angular regions to the ones of the other face. The imposition of boundary conditions yields a set of GWHEs for practical angular region problems.

For the sake of simplicity, even if challenging, this work is focused on an elastic wedge problem filled by an elastic isotropic solid and extendable to anisotropic media. This problem is considered a fundamental problem in the mathematical theory of elastic diffraction and, despite numerous attempts to solve it in closed form, no exact solution exists for arbitrary aperture angle of the wedge region. Three major semianalytical approaches [12–14] have been proposed to solve this problem in the two-dimensional case (i.e. at normal incidence). The first method is presented by Budaev in his monograph [12] that is based on the Sommerfeld integral representation of the elastic potentials and extends the popular and effective Sommerfeld–Malyuzhinets method to wedge problems with two concurrent different propagation constants. The difference equations, that initially arise from this formulation, are reduced to singular integral equations that are treated with a regularization method. Further interesting aspects of this formulation are presented also in [15]. A second method to study elastic wedge problems is reported in [13], where the scattered field by the faces of the wedge is related to the Fourier transforms of the displacement field of the faces (the spectral functions). Applying the Fourier transforms to the differential formulation of the elastic field and taking into account the boundary conditions, the authors obtain singular integral equations in terms of the spectral functions that are numerically solved by using the Galerkin collocation method. An important aspect of this work is the use of recursive equations that provide analytical continuation (propagation of the solution) of the approximate spectral functions obtained by the numerical solution in a certain strip. New developments of this method are reported in [16], where double Fourier transforms are introduced to obtain the kernels of the singular integral equations. In [17], the method is extended to three-dimensional problems, however, the proposed functional equations in spectral domain are again written in terms of singular integral operators and not in an algebraic form. The concept of spectral representation of the displacements on the wedge faces is applied also by Gautesen’s group works [14,18–20] that, according to our opinion, have produced the best practical results in the solution of the two-dimensional elastic isotropic wedge problem [14]. The difference with respect to [13] is the use of an integral representation in terms of the displacements in the natural domain. Substantially, the integral representations of this method are those that in electromagnetism are called Kirchhoff’s representations. The kernel of the integral representations are suitable Green functions of the free space and the integral does not contain components of the stress tensors. The traction-free boundary conditions on the faces of the wedge impose this property. Another important aspect in these works is to resort to an extinction theorem that allows to impose the vanishing of the displacement outside the elastic wedge. The application of the theorem allows to use unilateral Fourier transform (or Laplace transform) on the Gautesen (Kirchhoff) integral representations and it yields functional equations that are algebraic with respect to the Laplace transforms of the displacements on the two faces of the wedge. We note that the arguments of the Laplace transforms of the displacements on the two faces are different. Substantially, the functional equations obtained in [14] are GWHEs,^1^ although not defined in this way.

In this paper, we derive with a systematic and efficient method spectral functional equations in algebraic form useful to derive GWHEs in three-dimensional elastic wedge problems. These equations are validated by comparison with the ones proposed in [14]. The proposed method has the following important characteristics:

(i) The functional equations are easily obtained in terms of eigenvectors and eigenvalues of a matrix that characterizes the medium filling the angular region.
(ii) These functional equations hold independently from the boundary conditions of the angular region.
(iii) The application of boundary conditions yields a system of GWHEs for a specific problem.
(iv) The deduction of the GWHEs is general, since the method can be applied to study wave motion in angular regions filled by arbitrary linear media.

We remark that property (i) avoids the introduction of Kirchhoff-type representations that require the computation of Green’s function. This computation can be difficult in elasticity, see Gautesen’s group works [14]. Property (ii) allows the possibility to study complex wave motion problems constituted of different angular subregions or angular regions connected to planar stratified media, see in electromagnetics [21–24]. The third and the fourth characteristics allow the derivation of GWHEs in isotropic elastic media with plane wave source at skew incidence and in the general case of an elastic wedge filled by anisotropic medium. Moreover, we note that it is possible to directly compute from the spectral solution of the GWHEs the field in every point of the angular regions, avoiding Kirchhoff’s representations and Green’s function in natural domain. In particular, the diffracted field component can be asymptotically computed with the saddle point method. A last but not less important property of the GWHE formulations of wedge problems is constituted by the set of mathematical tools in complex analysis. The WH technique provides powerful solution methods based on exact and approximate factorization methods. In their works, Gautesen *et al*. have proposed a possible original method to deal with GWHEs of elastic wedge problems, exploiting analytical properties of the unknowns, see [14] and references therein. We propose, alternatively, the Fredholm factorization method [6,7], which is an effective semianalytical technique for the solution of arbitrary GWHEs and it is based on the reduction of the factorization problem to Fredholm integral equations of the second kind. We expect, in a future work, to effectively apply the Fredholm factorization to solve the GWHEs of elastic wedge problems using the same methodology applied in electromagnetic scattering from dielectric wedge [5,25–28].

The paper is organized into eight sections and we assume plane wave sources and/or sources localized at infinity in time-harmonic fields with a time dependence specified by $e^{j\omega t}$ (electrical engineering notation) that is suppressed. In §2, we introduce the first-order vector differential formulation for continuous components of the elastic field in an indefinite homogeneous medium. Note that, while in electromagnetics the continuous components of field are the transversal ones, in elasticity, we have a more complex definition in terms of stress tensor and velocity vector. The theory presented in §2 is also useful to study propagation in stratified media. Using oblique Cartesian coordinates and taking into account the results of §2, §3 describes the novel application of the method to angular regions, yielding the oblique first-order vector differential formulation for continuous components of the elastic field. The application of Laplace transform along one face of the angular region and assumption of a problem with invariance along the edge profile yield a vector ordinary differential problem of first order (oblique equations). The solution of these oblique equations, projected on the reciprocal vectors of an algebraic matrix defined in §2, provides the functional equations of an arbitrary angular region (§4). It is remarkable that we get functional equations independently from the materials and the sources that can be present outside of the considered angular region. Explicit expressions in algebraic form are reported in §5 for isotropic media and arbitrary boundary conditions. Section 6 shows the validation of functional equations in special simplified cases reported in literature by other authors for the planar problem; and the electronic supplementary material reports the validation of functional equations by evaluating the characteristic impedances of half spaces in planar problems. Finally, conclusions are reported in §7 and a glossary of the symbols useful for the readability of the text is provided at the end (table 2). We remark that, according to our opinion, the functional equations for the non-planar (three-dimensional) general case are deduced and reported for the first time in literature in this paper in §5. We finally state that the scope of our paper is to present algebraic spectral functional equations for arbitrary boundary conditions for three-dimensional wave motion problems in angular regions that are useful for the examination of practical problems by imposing specific boundary conditions yielding GWHE formulations.

## 2. First-order differential equations for continuous components of the elastic field in an indefinite rectangular isotropic medium

In this section, we study elastic wave propagation in stratified media along a direction (say y) and, consequently in §3, we use these results to develop the theory for angular regions.

The evaluation of the physical fields in an elastic linear medium can be generally described by a system of partial differential equations of first order. In the absence of sources localized at finite or in the presence of plane wave sources, the system is constituted of the translational equation of motion and the stress–displacement equation [29,30], i.e. considering dydadic notation and time harmonic regime we have

2.1 $$\nabla \cdot \underline{T}=-\rho \omega^{2}\text{u}$$

and

2.2 $$\underline{S}=\frac{1}{2}(\nabla \text{u}+{\left(\nabla \text{u}\right)}^{\prime }),$$

where $\underline{T}$, $\underline{S}$ and u are, respectively, the stress tensor, the strain tensor and the displacement vector, and ρ is the mass density (${\;}^{\prime }$ stands for transpose). In a general media, the stress and strain tensors have a constitutive relation given by Hooke’s Law

2.3 $$\underline{T}=\underline{\underline{C}}:\underline{S},$$

where $\underline{\underline{C}}$ is a fourth-order stiffness tensor that in isotropic media simplifies to

2.4 $$\underline{\underline{C}}=\lambda \underline{I}\;\underline{I}+2\mu {\underline{\underline{I}}}^{\mathrm{sym}},$$

where λ and μ are Lamé’s constants of the elastic medium and, $\underline{I}$ and ${\underline{\underline{I}}}^{\mathrm{sym}}$ are, respectively, the unit dyadic and the symmetric fourth-order unit dyadic (tetradic).

Using vector (Voigt) representation for tensor quantities [29] we rewrite (2.1) as

2.5 $$\nabla_{T}\text{T}=j\omega \;\text{p}$$

and

2.6 $$\nabla_{v}\text{v}=j\omega \;\text{S},$$

with

2.7 $$\nabla_{T}=\left(\begin{array}{cccccc} \frac{\partial }{\partial x} & 0 & 0 & 0 & \frac{\partial }{\partial z} & \frac{\partial }{\partial y}\\ 0 & \frac{\partial }{\partial y} & 0 & \frac{\partial }{\partial z} & 0 & \frac{\partial }{\partial x}\\ 0 & 0 & \frac{\partial }{\partial z} & \frac{\partial }{\partial y} & \frac{\partial }{\partial x} & 0 \end{array}\right)\;\text{and}\;\nabla_{v}=\left(\begin{array}{ccc} \frac{\partial }{\partial x} & 0 & 0\\ 0 & \frac{\partial }{\partial y} & 0\\ 0 & 0 & \frac{\partial }{\partial z}\\ 0 & \frac{\partial }{\partial z} & \frac{\partial }{\partial y}\\ \frac{\partial }{\partial z} & 0 & \frac{\partial }{\partial x}\\ \frac{\partial }{\partial y} & \frac{\partial }{\partial x} & 0 \end{array}\right)={\left(\nabla_{T}\right)}^{\prime },$$

and where T, S, p and v are, respectively, the symmetric stress tensor in six-component vector form (2.8), the symmetric strain tensor in six-component vector form (2.8), the vector momentum density p=ρv and the vector particle velocity v=jωu:

2.8 $$\text{T}={\left(T_{xx},T_{yy},T_{zz},T_{yz},T_{xz},T_{xy}\right)}^{\prime }\;\text{and}\;\text{S}={\left(S_{xx},S_{yy},S_{zz},2S_{yz},2S_{xz},2S_{xy}\right)}^{\prime }.$$

Inspired by [1], for electromagnetic applications, to effectively study wave motion problems in elasticity, it is convenient to introduce the concept of transverse equations using abstract notation.

The homogeneous abstract form of (2.5) and (2.6), see §2.9 of [4], is

2.9 $$\Gamma_{\nabla }\;\psi =j\omega \theta ,$$

where $\Gamma_{\nabla }$ is a matrix differential operator of first order that relates the fields ψ and θ:

2.10 $$\psi =\left(\begin{array}{c} \text{T}\\ \text{v} \end{array}\right),\;\theta =\left(\begin{array}{c} \text{S}\\ \text{p} \end{array}\right),\;\Gamma_{\nabla }=\left(\begin{array}{cc} 0 & \nabla_{v}\\ \nabla_{T} & 0 \end{array}\right).$$

The vectors ψ and θ have a constitutive relation defined by the equation

2.11 θ=W ψ,

where the matrix W depends on the medium that is considered.

In order to close the mathematical problem (2.9)–(2.11), we need to enforce the geometrical domain of the problem, its boundaries' conditions and the radiation condition.

For simplicity, in the following, we consider isotropic loss-less material, however we claim that transversal elastic equations in a general anisotropic medium assume the same form. Considering Hooke’s Law T=CS in a loss-less isotropic medium we obtain

2.12 $$W=\left(\begin{array}{cc} C^{-1} & O\\ O & R \end{array}\right),\;C=\left(\begin{array}{cccccc} \lambda +2\mu & \lambda & \lambda & 0 & 0 & 0\\ \lambda & \lambda +2\mu & \lambda & 0 & 0 & 0\\ \lambda & \lambda & \lambda +2\mu & 0 & 0 & 0\\ 0 & 0 & 0 & \mu & 0 & 0\\ 0 & 0 & 0 & 0 & \mu & 0\\ 0 & 0 & 0 & 0 & 0 & \mu \end{array}\right),\;R=\left(\begin{array}{ccc} \rho & 0 & 0\\ 0 & \rho & 0\\ 0 & 0 & \rho \end{array}\right).$$

In the following, we use also alternative parameters to define the medium characteristics with respect to the mass density ρ, and Lamé’s constants λ and μ

2.13 $$k_{p}=\omega \sqrt{\frac{\rho }{\lambda +2\mu }},\;k_{s}=\omega \sqrt{\frac{\rho }{\mu }}\;\text{and}\;Z_{o}^{\;}=\frac{k_{s}\mu }{\omega },$$

where $k_{p}$ is the propagation constant of the longitudinal/principal wave, $k_{s}$ is the propagation constant of the transversal/secondary wave (vertical or horizontal) and the impedance $Z_{o}$ is a quantity such that stress components have the same dimensions of velocity components time $Z_{o}$.

Comparing equations (2.9)–(2.12) to the ones reported in [1] for electromagnetic applications, we note that the stress T, the particle velocity v, the strain S and the momentum density p are analogous, respectively, to the electric field E, the magnetic field H, the dielectric induction D and the magnetic induction B with constitutive relations T=CS and p=ρv analogous, respectively, to $\text{E}=\varepsilon^{-1}\text{D}$ and B=μH (where ε, μ can be either scalar or tensor). Moreover, (2.5)–(2.6) are the elastic analogue of Maxwell’s equations in electromagnetism.

Substituting (2.11) into (2.9) with (2.12)–(2.13) we get the nine equations that relate the stress T with the velocity v [4]:

2.14 $$(\Gamma_{\nabla }-j\omega W)\;\psi =\text{ 0},$$

whose explicit form is

2.15  DxTxx+DzTxz+DyTxy=jks Zovx, DyTyy+DzTyz+DxTxy=jks Zovy, DzTzz+DyTyz+DxTxz=jks Zovz, Dxvx=jks [2kp2(Txx−Tyy−Tzz)+ks2(−2Txx+Tyy+Tzz)]8kp2Zo−6ks2Zo, Dyvy=jks [ks2(Txx−2Tyy+Tzz)−2kp2(Txx−Tyy+Tzz)]8kp2Zo−6ks2Zo, Dzvz=jks [ks2(Txx+Tyy−2Tzz)−2kp2(Txx+Tyy−Tzz)]8kp2Zo−6ks2Zo Dzvy+Dyvz=jks TyzZo, Dzvx+Dxvz=jks TxzZoandDyvx+Dxvy=jks TxyZo}

where $D_{x}=\partial /\partial x$, $D_{y}=\partial /\partial y$, $D_{z}=\partial /\partial z$.

While the constitutive parameters change only in one direction, say y, using the divergence theorem [29], it is possible to demonstrate that the continuous components of ψ at interfaces are the ones of v and $n\cdot \underline{T}$, where n is the unit normal at the interface, i.e.

2.16 $$\psi_{t}={\left(T_{yy},T_{yz},T_{xy},v_{x},v_{y},v_{z}\right)}^{\prime }.$$

The transverse equations of a field are equations that involve only the components that remain continuous along the stratification according to the boundary conditions on the interfaces and, starting from (2.15), in general they assume the following form:

2.17 −∂∂yψt=M(∂∂z,∂∂x) ψt,

where we have a first-order derivative along y and a matrix differential operator in x and z.

The reduction of the elastic differential problems to the transverse equations starts from deriving expressions of the discontinuous components (along y) direction ($T_{xx},T_{zz},T_{xz}$) from the fourth, the sixth and the eighth of (2.15). We get

2.18 $$\begin{array}{rl} & T_{xx}=\frac{{k_{p}}^{2}(-2k_{s}T_{yy}+4jZ_{o}(D_{x}v_{x}+D_{z}v_{z}))+{k_{s}}^{2}(k_{s}T_{yy}-2jZ_{o}(2D_{x}v_{x}+D_{z}v_{z}))}{{k_{s}}^{3}},\\ & T_{zz}=\frac{{k_{p}}^{2}(-2k_{s}T_{yy}+4jZ_{o}(D_{x}v_{x}+D_{z}v_{z}))+{k_{s}}^{2}(k_{s}T_{yy}-2jZ_{o}(D_{x}v_{x}+2D_{z}v_{z}))}{{k_{s}}^{3}}\\ \text{and}\;\; & T_{xz}=-\frac{j(D_{z}v_{x}+D_{x}v_{z})Z_{o}}{k_{s}} \end{array}\}$$

By substituting (2.18) into the six non-used equations of (2.15) (i.e. equations at lines 1, 2, 3, 5, 7 and 9) we get the transverse equations (2.17), where

2.19 $$\begin{array}{rl} & M\left(\frac{\partial }{\partial z},\frac{\partial }{\partial x}\right)\\ & \;\;=\left(\begin{array}{cccccc} 0 & D_{z} & D_{x} & 0 & -jk_{s}Z_{o} & 0\\ D_{z}-\frac{2D_{z}{k_{p}}^{2}}{{k_{s}}^{2}} & 0 & 0 & \frac{jD_{x}D_{z}(4{k_{p}}^{2}-3{k_{s}}^{2})Z_{o}}{{k_{s}}^{3}} & 0 & M_{26}(D_{z},D_{x})\\ D_{x}-\frac{2D_{x}{k_{p}}^{2}}{{k_{s}}^{2}} & 0 & 0 & M_{34}(D_{z},D_{x}) & 0 & \frac{jD_{x}D_{z}(4{k_{p}}^{2}-3{k_{s}}^{2})Z_{o}}{{k_{s}}^{3}}\\ 0 & 0 & -\frac{jk_{s}}{Z_{o}} & 0 & D_{x} & 0\\ -\frac{j{k_{p}}^{2}}{k_{s}Z_{o}} & 0 & 0 & D_{x}-\frac{2D_{x}{k_{p}}^{2}}{{k_{s}}^{2}} & 0 & D_{z}-\frac{2D_{z}{k_{p}}^{2}}{{k_{s}}^{2}}\\ 0 & -\frac{jk_{s}}{Z_{o}} & 0 & 0 & D_{z} & 0 \end{array}\right), \end{array}$$

2.20 $$\begin{array}{rl} & M_{34}(D_{z},D_{x})=-\frac{j({k_{s}}^{4}+(4{D_{x}}^{2}+{D_{z}}^{2}){k_{s}}^{2}-4{D_{x}}^{2}{k_{p}}^{2})Z_{o}}{{k_{s}}^{3}} \end{array}$$

2.21 $$\begin{array}{rl} \text{and}\;\; & M_{26}(D_{z},D_{x})=-\frac{j({k_{s}}^{4}+({D_{x}}^{2}+4{D_{z}}^{2}){k_{s}}^{2}-4{D_{z}}^{2}{k_{p}}^{2})Z_{o}}{{k_{s}}^{3}}, \end{array}$$

and where $D_{x}=\partial /\partial x$, $D_{y}=\partial /\partial y$, $D_{z}=\partial /\partial z$.

The transverse equations along the y-direction take the form reported in (2.17), where M(∂/∂z,∂/∂x) is a matrix differential operator of arbitrary differential order and dimension that, in the case of electromagnetic and elastic problems, has, respectively, dimensions 4 and 6, both with differential order 2 in x and z. In the following, we assume that the geometry of the elastic wave-motion problem as well as the eventual boundary conditions are invariant along the z-direction, thus, without loss of generality, when a source depends on an $e^{-j\alpha_{o}\;z}$ factor, also the total field depends on the same factor, i.e. $\psi_{t}=\psi_{t}(x,y,z)=\text{f}(x,y)\;e^{-j\alpha_{o}\;z}$, see for instance [17] before (2.8). Of course, the same behaviour can be obtained by applying a Fourier transform also along the z-direction and assuming an incident plane wave with a particular skew direction that yields $e^{-j\alpha_{o}\;z}$. However, for simplicity, we prefer to avoid the use of a double Fourier transform, recalling that in the present context an arbitrary source can be expanded in a summation of plane waves.

It yields $\left(\partial /\partial z)\psi_{t}(x,y,z)=-j\alpha_{o}\psi_{t}(x,y,z\right)$, i.e. $\partial /\partial z\to -j\alpha_{o}$, thus

2.22 $$M\left(\frac{\partial }{\partial z},\frac{\partial }{\partial x}\right)=M\left(-j\alpha_{o},\frac{\partial }{\partial x}\right)=M_{o}+M_{1}\frac{\partial }{\partial x}+M_{2}\frac{\partial^{2}}{\partial x^{2}},$$

where $M_{m}$ with m=0,1,2 are 6×6 matrices and do not depend on x, as they are easily derived from (2.19)

2.23 $$\begin{array}{rl} & M_{o}=\left(\begin{array}{cccccc} 0 & -j\alpha_{o} & 0 & 0 & -jk_{s}Z_{o} & 0\\ -j\alpha_{o}\left(1-\frac{2{k_{p}}^{2}}{{k_{s}}^{2}}\right) & 0 & 0 & 0 & 0 & -\frac{jZ_{o}(4{\alpha_{o}}^{2}{k_{p}}^{2}+{k_{s}}^{4}-4{\alpha_{o}}^{2}{k_{s}}^{2})}{{k_{s}}^{3}}\\ 0 & 0 & 0 & -\frac{jZ_{o}({k_{s}}^{2}-{\alpha_{o}}^{2})}{k_{s}} & 0 & 0\\ 0 & 0 & -\frac{jk_{s}}{Z_{o}} & 0 & 0 & 0\\ -\frac{j{k_{p}}^{2}}{k_{s}Z_{o}} & 0 & 0 & 0 & 0 & -j\alpha_{o}\left(1-\frac{2{k_{p}}^{2}}{{k_{s}}^{2}}\right)\\ 0 & -\frac{jk_{s}}{Z_{o}} & 0 & 0 & -j\alpha_{o} & 0 \end{array}\right), \end{array}$$

2.24 $$\begin{array}{rl} & M_{1}=\left(\begin{array}{cccccc} 0 & 0 & 1 & 0 & 0 & 0\\ 0 & 0 & 0 & \frac{\alpha_{o}Z_{o}(4{k_{p}}^{2}-3{k_{s}}^{2})}{{k_{s}}^{3}} & 0 & 0\\ 1-\frac{2{k_{p}}^{2}}{{k_{s}}^{2}} & 0 & 0 & 0 & 0 & \frac{\alpha_{o}Z_{o}(4{k_{p}}^{2}-3{k_{s}}^{2})}{{k_{s}}^{3}}\\ 0 & 0 & 0 & 0 & 1 & 0\\ 0 & 0 & 0 & 1-\frac{2{k_{p}}^{2}}{{k_{s}}^{2}} & 0 & 0\\ 0 & 0 & 0 & 0 & 0 & 0 \end{array}\right) \end{array}$$

2.25 $$\begin{array}{rl} \text{and}\; & M_{2}=\left(\begin{array}{cccccc} 0 & 0 & 0 & 0 & 0 & 0\\ 0 & 0 & 0 & 0 & 0 & -\frac{jZ_{o}}{k_{s}}\\ 0 & 0 & 0 & \frac{4jZ_{o}({k_{p}}^{2}-{k_{s}}^{2})}{{k_{s}}^{3}} & 0 & 0\\ 0 & 0 & 0 & 0 & 0 & 0\\ 0 & 0 & 0 & 0 & 0 & 0\\ 0 & 0 & 0 & 0 & 0 & 0 \end{array}\right). \end{array}$$

### (a) The eigenvalues and the eigenvectors of M in spectral domain

By applying a Fourier transform along the x-direction to (2.17) with (2.22)–(2.25) ($M_{m}=0,\;m>2$) in the absence of source, we obtain an ordinary vector first-order differential equation

2.26 $$-\frac{\text{d}}{\text{d}y}\Psi_{t}(\eta )=M(\eta )\;\Psi_{t}(\eta ),$$

where $\psi_{t}(x)=(1/2\pi )\int_{-\infty }^{\infty }\Psi_{t}(\eta )e^{-j\eta x}\;d\eta$ (notation with omission of y, z dependence) and

2.27 $$M(\eta )=M(-j\alpha_{o},-j\eta )=M_{o}-j\eta M_{1}-\eta^{2}M_{2},$$

where $\frac{\partial }{\partial z}\to -j\alpha_{o}$ for the field factor $e^{-j\alpha_{o}\;z}$ (see comment before (2.22)) and $\frac{\partial }{\partial x}\to -j\eta$ for the property of Fourier transforms.

Now, let us investigate the properties of the eigenvalue problem (2.28) associated with (2.26)

2.28 $$M(\eta )\;{\text{u}}_{i}(\eta )=\lambda_{i}(\eta ){\text{u}}_{i}(\eta ),$$

where ${\text{u}}_{i}(\eta )$ and $\lambda_{i}$ (i=1…n) are, respectively, the eigenvectors and the eigenvalues of the 6×6 matrix M(η) (2.27). In the presence of a passive medium, we observe that three eigenvalues (say $\lambda_{1},\lambda_{2},\lambda_{3}$) present non-negative real part and the other three eigenvalues (say $\lambda_{4},\lambda_{5},\lambda_{6}$) present non-positive real part. In the following, we use also alternative expressions:

2.29 $$\lambda_{1}=j\xi_{p}(\eta )=-\lambda_{4},\;\lambda_{2}=\lambda_{3}=j\xi_{s}(\eta )=-\lambda_{5}=-\lambda_{6}.$$

The explicit form of (2.29) are expressed in terms of $\tau_{op}=\sqrt{k_{p}^{2}-\alpha_{o}^{2}}$, $\tau_{os}=\sqrt{k_{s}^{2}-\alpha_{o}^{2}}$

2.30 $$\xi_{p}(\eta )=\sqrt{\tau_{op}^{2}-\eta^{2}},\;\xi_{s}(\eta )=\sqrt{\tau_{os}^{2}-\eta^{2}},$$

with $Im[k_{p,s}]<0$, $Im[\tau_{op,os}]<0$ in lossy media. Since $k_{p,s}^{2}=k_{x}^{2}+k_{y}^{2}+k_{z}^{2}=\eta^{2}+\xi_{p,s}^{2}+\alpha_{o}^{2}$, $\xi_{p,s}(\eta )$ are multivalued functions of η. In the following, we assume as proper sheets of $\xi_{p,s}(\eta )$, the ones with $\xi_{p,s}(0)=\tau_{op,os}$ and as branch lines of $\xi_{p,s}(\eta )$ the classical line $Im[\xi_{p,s}(\eta )]=0$ (see in practical engineering estimations Ch. 5.3b of [31]) or the vertical line $\left(Re[\eta ]=Re[\tau_{os,op}],\;Im[\eta ]<Im[\tau_{os,op}]\right)$. In (2.29), we have that $\lambda_{1},\lambda_{2},\lambda_{3}$ ($\lambda_{4},\;\lambda_{5},\;\lambda_{6}$) are related to progressive (regressive) waves and, $\xi_{p,s}$ are with non-positive imaginary part. In this framework, we associate the direction of propagation to attenuation phenomena.

Since the matrix M(η) is diagonalizable, M(η) is semisimple^2^ ([32], Ch. V.9). The semisimple property is fundamental to develop the procedure as it yields a set of independent eigenvectors ${\text{u}}_{i}(\eta )$ even with the same eigenvalues. Although a theory about geometric and mathematical multiplicity of eigenvalues is available in practice, we checked the diagonalizability of M(η) using a Jordan decomposition algorithm that in our case yields $M(\eta )=U^{-1}DU$, where the matrix U is a matrix with column elements ${\text{u}}_{i}(\eta )$ and D is a diagonal matrix with diagonal elements the eigenvalues $\lambda_{i}$. In relation to the eigenvectors ${\text{u}}_{i}(\eta )$, we introduce the reciprocal vectors $\nu_{i}(\eta )$ (see ch. 3.16 of [32]) that, in the general elastic case with $\alpha_{o}\neq 0$, can be computed by inversion of the matrix U. The vectors $\nu_{i}(\eta )$ satisfy the bi-orthogonal relations

2.31 $$\nu_{j}\cdot {\text{u}}_{i}=\delta_{ji},\;i.e.\;{\underline{1}}_{t}=\sum_{i=1}^{6}{\text{u}}_{i}\nu_{i},$$

where ⋅ is the vector scalar product, $\delta_{ij}$ is the Kronecker symbol and ${\underline{1}}_{t}$ is the unit dyadic defined in terms of dyadic products and such that ${\underline{1}}_{t}\cdot \text{a}=\text{a}\cdot {\underline{1}}_{t}=\text{a}$ for an arbitrary vector a.

From a physics point of view, the eigenvalues $\lambda_{1}=-\lambda_{4}$ are associated with longitudinal P (principal) waves, while $\lambda_{2}=-\lambda_{5}$ and $\lambda_{3}=-\lambda_{6}$ are relevant to the transversal S (secondary) waves of two types: secondary vertical (SV) and secondary horizontal (SH). The P, SV and SH waves are not decoupled when $\alpha_{o}\neq 0$, while if $\alpha_{o}=0$ we have two decoupled problems: one related to P and SV waves (planar problem) and the other to SH waves (anti-planar problem).

The computation of eigenvectors in (2.28), using Wolfram Mathematica®, it yields in compact notation

2.32 $$U=\left(\begin{array}{cccccc} \frac{Z_{o}({\alpha_{o}}^{2}+\eta^{2}-\xi_{s}^{2})}{k_{s}\alpha_{o}} & -\frac{2Z_{o}\xi_{s}}{k_{s}} & 0 & \frac{Z_{o}({\alpha_{o}}^{2}+\eta^{2}-\xi_{s}^{2})}{k_{s}\alpha_{o}} & \frac{2Z_{o}\xi_{s}}{k_{s}} & 0\\ -\frac{2Z_{o}\xi_{p}}{k_{s}} & -\frac{\alpha_{o}Z_{o}}{k_{s}} & -\frac{Z_{o}\xi_{s}}{k_{s}} & \frac{2Z_{o}\xi_{p}}{k_{s}} & -\frac{\alpha_{o}Z_{o}}{k_{s}} & \frac{Z_{o}\xi_{s}}{k_{s}}\\ -\frac{2\eta Z_{o}\xi_{p}}{k_{s}\alpha_{o}} & \frac{Z_{o}(\xi_{s}^{2}-\eta^{2})}{k_{s}\eta } & \frac{\alpha_{o}Z_{o}\xi_{s}}{k_{s}\eta } & \frac{2\eta Z_{o}\xi_{p}}{k_{s}\alpha_{o}} & \frac{Z_{o}(\xi_{s}^{2}-\eta^{2})}{k_{s}\eta } & -\frac{\alpha_{o}Z_{o}\xi_{s}}{k_{s}\eta }\\ \frac{\eta }{\alpha_{o}} & -\frac{\xi_{s}}{\eta } & -\frac{\alpha_{o}}{\eta } & \frac{\eta }{\alpha_{o}} & \frac{\xi_{s}}{\eta } & -\frac{\alpha_{o}}{\eta }\\ \frac{\xi_{p}}{\alpha_{o}} & 1 & 0 & -\frac{\xi_{p}}{\alpha_{o}} & 1 & 0\\ 1 & 0 & 1 & 1 & 0 & 1 \end{array}\right),$$

whose columns are ${\text{u}}_{i}(\eta )$ corresponding to the eigenvalues as defined and ordered in (2.29). The inverse of U yields in its rows the reciprocal vectors $\nu_{i}(\eta )$

2.33 $$V=\left(\begin{array}{cccccc} -\frac{\alpha_{o}}{2k_{s}Z_{o}} & -\frac{{\alpha_{o}}^{2}}{2k_{s}Z_{o}\xi_{p}} & -\frac{\alpha_{o}\eta }{2k_{s}Z_{o}\xi_{p}} & \frac{\alpha_{o}\eta }{{k_{s}}^{2}} & -\frac{\alpha_{o}({\alpha_{o}}^{2}+\eta^{2}-\xi_{s}^{2})}{2{k_{s}}^{2}\xi_{p}} & \frac{{\alpha_{o}}^{2}}{{k_{s}}^{2}}\\ -\frac{{\alpha_{o}}^{2}+\eta^{2}}{2k_{s}Z_{o}\xi_{s}} & \frac{\alpha_{o}}{2k_{s}Z_{o}} & \frac{\eta }{2k_{s}Z_{o}} & \frac{\eta ({\alpha_{o}}^{2}+\eta^{2}-\xi_{s}^{2})}{2{k_{s}}^{2}\xi_{s}} & \frac{{\alpha_{o}}^{2}+\eta^{2}}{{k_{s}}^{2}} & \frac{\alpha_{o}({\alpha_{o}}^{2}+\eta^{2}-\xi_{s}^{2})}{2{k_{s}}^{2}\xi_{s}}\\ \frac{\alpha_{o}}{2k_{s}Z_{o}} & -\frac{\left(k_{s}-\alpha_{o})(k_{s}+\alpha_{o}\right)}{2k_{s}Z_{o}\xi_{s}} & \frac{\alpha_{o}\eta }{2k_{s}Z_{o}\xi_{s}} & -\frac{\alpha_{o}\eta }{{k_{s}}^{2}} & -\frac{\alpha_{o}\xi_{s}}{{k_{s}}^{2}} & \frac{1}{2}-\frac{{\alpha_{o}}^{2}}{{k_{s}}^{2}}\\ -\frac{\alpha_{o}}{2k_{s}Z_{o}} & \frac{{\alpha_{o}}^{2}}{2k_{s}Z_{o}\xi_{p}} & \frac{\alpha_{o}\eta }{2k_{s}Z_{o}\xi_{p}} & \frac{\alpha_{o}\eta }{{k_{s}}^{2}} & \frac{\alpha_{o}({\alpha_{o}}^{2}+\eta^{2}-\xi_{s}^{2})}{2{k_{s}}^{2}\xi_{p}} & \frac{{\alpha_{o}}^{2}}{{k_{s}}^{2}}\\ \frac{{\alpha_{o}}^{2}+\eta^{2}}{2k_{s}Z_{o}\xi_{s}} & \frac{\alpha_{o}}{2k_{s}Z_{o}} & \frac{\eta }{2k_{s}Z_{o}} & -\frac{\eta ({\alpha_{o}}^{2}+\eta^{2}-\xi_{s}^{2})}{2{k_{s}}^{2}\xi_{s}} & \frac{{\alpha_{o}}^{2}+\eta^{2}}{{k_{s}}^{2}} & -\frac{\alpha_{o}({\alpha_{o}}^{2}+\eta^{2}-\xi_{s}^{2})}{2{k_{s}}^{2}\xi_{s}}\\ \frac{\alpha_{o}}{2k_{s}Z_{o}} & \frac{\left(k_{s}-\alpha_{o})(k_{s}+\alpha_{o}\right)}{2k_{s}Z_{o}\xi_{s}} & -\frac{\alpha_{o}\eta }{2k_{s}Z_{o}\xi_{s}} & -\frac{\alpha_{o}\eta }{{k_{s}}^{2}} & \frac{\alpha_{o}\xi_{s}}{{k_{s}}^{2}} & \frac{1}{2}-\frac{{\alpha_{o}}^{2}}{{k_{s}}^{2}} \end{array}\right).$$

In the following §§3–5, the eigenvectors ${\text{u}}_{i}(\eta )$ and the reciprocal vectors $\nu_{i}(\eta )$ will be used to obtain functional equations that relate spectral quantities in elastic wave motion problems between the two terminal faces of an angular region for an arbitrary $\alpha_{o}$, i.e. non-planar problems. We also note that ${\text{u}}_{i}(\eta )$ and $\nu_{i}(\eta )$ can be used to build the solution of the transverse equations (2.26) in Laplace domain for elastic wave motion problems in a rectangular stratified region [33].

## 3. First-order differential oblique equations for continuous components of the elastic field in an angular region

In this section, we introduce the oblique equations for continuous components of the elastic field in an angular region using an oblique system of Cartesian axes and applying the properties reported in §2 for rectangular regions. In the following sections, first, we deduce spectral functional equations then, by imposing boundary conditions, the GWHEs for angular shaped regions.

**Figure 1.**
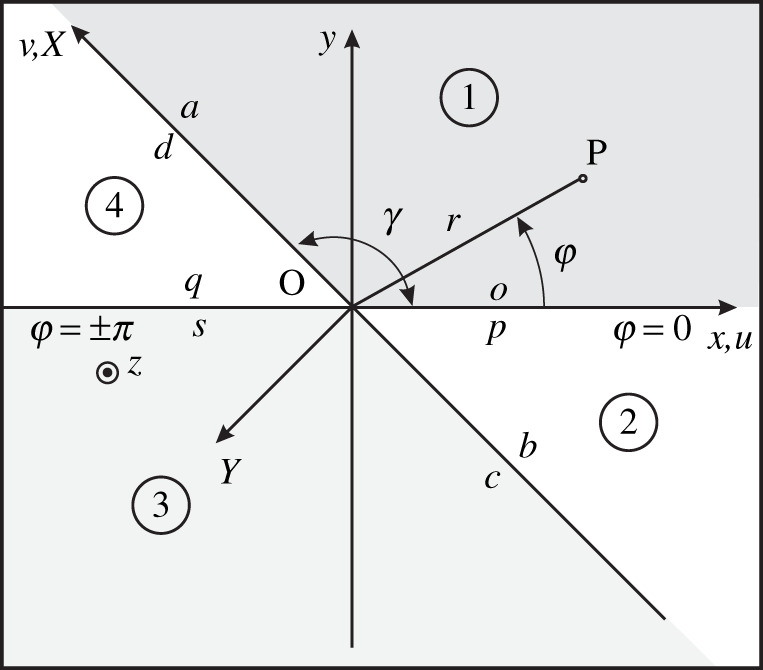
Angular regions and oblique Cartesian coordinates. The figure reports the x,y,z Cartesian coordinates and r,φ,z cylindrical coordinates useful to define the oblique Cartesian coordinate system u,v,z with reference to the angular region 1 0<φ<γ with 0<γ<π. In the figure, the space is divided into four angular regions delimited by φ=±γ,0,π, and the face boundaries are labelled a,b,c,d,o,p,q,s. The figure reports also the local-to-face-a Cartesian coordinate system X,Y,Z≡z. Note that x≡u and v≡X.

With reference to figure 1 where angular regions are defined thorough the angle γ (0<γ<π), we introduce the oblique Cartesian coordinates u,v,z in terms of the Cartesian coordinates x,y,z:

3.1 $$u=x-y\;\cot\gamma ,\;v=\frac{y}{\sin\gamma }\;\text{or}\;x=u+v\;\cos\gamma ,\;y=v\sin\gamma ,$$

with partial derivatives

3.2 $$\begin{array}{rl} & \frac{\partial }{\partial x}=\frac{\partial u}{\partial x}\frac{\partial }{\partial u}+\frac{\partial v}{\partial x}\frac{\partial }{\partial v}=\frac{\partial }{\partial u},\;\frac{\partial }{\partial y}=\frac{\partial u}{\partial y}\frac{\partial }{\partial u}+\frac{\partial v}{\partial y}\frac{\partial }{\partial v}=-\cot\gamma \;\frac{\partial }{\partial u}+\frac{1}{\sin\gamma }\;\frac{\partial }{\partial v}\\ \text{and}\;\; & \frac{\partial }{\partial u}=\frac{\partial x}{\partial u}\frac{\partial }{\partial x}+\frac{\partial y}{\partial u}\frac{\partial }{\partial y}=\frac{\partial }{\partial x},\;\frac{\partial }{\partial v}=\frac{\partial x}{\partial v}\frac{\partial }{\partial x}+\frac{\partial y}{\partial v}\frac{\partial }{\partial y}=\cos\gamma \;\frac{\partial }{\partial x}+\sin\gamma \;\frac{\partial }{\partial y}. \end{array}\}$$

Starting from (2.17) with (2.22) the transverse (with respect to y) equation of dimension n=6 for an elastic problem with invariant geometry along z-direction (i.e. $e^{-j\alpha_{o}z}$) is

3.3 $$-\frac{\partial }{\partial y}\psi_{t}=M\left(-j\alpha_{o},\frac{\partial }{\partial x}\right)\;\psi_{t}=\left(M_{o}+M_{1}\frac{\partial }{\partial x}+M_{2}\frac{\partial^{2}}{\partial x^{2}}\right)\;\psi_{t}.$$

Note that for elastic problems, we have second differential order in x. Substituting (3.2), in particular ∂/∂x=∂/∂u and ∂/∂y=−cot⁡γ(∂/∂u)+1/sin⁡γ(∂/∂v), into (3.3), we obtain

3.4 $$-\frac{\partial }{\partial v}\psi_{t}=M_{e}\left(-j\alpha_{o},\frac{\partial }{\partial u}\right)\;\psi_{t}=\left(M_{eo}+M_{e1}\frac{\partial }{\partial u}+M_{e2}\frac{\partial^{2}}{\partial u^{2}}\right)\;\psi_{t},$$

where

3.5 $$M_{eo}=M_{o}\sin\gamma_{\;},\;M_{e1}=M_{1}\sin\gamma_{\;}-I\cos\gamma_{\;},\;M_{e2}=M_{2}\sin\gamma_{\;}.$$

For the sake of simplicity and in order to get simple explicit expressions, we consider homogeneous isotropic media filling the angular regions. In this case, the explicit forms of $M_{em},\;m=0,1,2$ (3.5) are straightforwardly derived from (2.23)–(2.25). By applying the Fourier transform along x=u direction to (3.4), i.e. $\psi_{t}(x)=(1/2\pi )\int_{-\infty }^{\infty }\Psi_{t}(\eta )e^{-j\eta x}\;d\eta$ with notation omitting v,z dependence, we obtain the ordinary system of differential equations

3.6 $$-\frac{\partial }{\partial v}\Psi_{t}=M_{e}(\gamma ,\eta )\;\Psi_{t}$$

with

3.7 $$M_{e}(\gamma ,\eta )=M_{e}(-j\alpha_{o},-j\eta )=M_{eo}-j\eta M_{e1}-\eta^{2}M_{e2}$$

since $\partial /\partial u=(\partial /\partial x)\overset{FT}{↔}-j\eta$.

### (a) Link between eigenvalues of M(η) and $M_{e}(\gamma ,\eta )$

In the oblique coordinate system, the solution of (3.6) is related to the eigenvalue problem

3.8 $$M_{e}(\gamma ,\eta )\;{\text{u}}_{ei}(\gamma ,\eta )=\lambda_{ei}(\gamma ,\eta ){\text{u}}_{ei}(\gamma ,\eta ),$$

where $\lambda_{ei}$ and ${\text{u}}_{ei}(\gamma ,\eta )$ (i=1…n) are, respectively, the eigenvalues and the eigenvectors of the 6×6 matrix $M_{e}(\gamma ,\eta )$. Using (3.6) and (3.7), equation (3.8) becomes

3.9 $$\left(M_{o}\sin\gamma_{\;}-j\eta M_{1}\sin\gamma -\eta^{2}M_{2}\sin\gamma_{\;})\;{\text{u}}_{ei}(\gamma ,\eta )=(\lambda_{ei}(\gamma ,\eta )-j\eta \cos\gamma ){\text{u}}_{ei}(\gamma ,\eta \right)$$

and thus

3.10 $$M(\eta )\;{\text{u}}_{ei}(\gamma ,\eta )=\left(\frac{\lambda_{ei}(\gamma ,\eta )-j\eta \cos\gamma }{\sin\gamma }\right){\text{u}}_{ei}(\gamma ,\eta ).$$

Comparing (3.10) with (2.28), we observe the relation among the eigenvalues and the eigenvectors of the two problems. The two problems defined by the matrices M(η) and $M_{e}(\gamma ,\eta )$ have the same eigenvectors

3.11 $${\text{u}}_{ei}(\gamma ,\eta )={\text{u}}_{i}(\eta ),$$

thus the same reciprocal vectors and related eigenvalues

3.12 $$\frac{\lambda_{ei}(\gamma ,\eta )-j\eta \cos\gamma }{\sin\gamma }=\lambda_{i}(\eta ).$$

Since $M_{e}(\gamma ,\eta )$ and M(η) have the same eigenvectors (3.11), i.e. ${\text{u}}_{i}(\eta )$ reported in the columns of (2.32), we note the important property that the eigenvectors of $M_{e}(\gamma ,\eta )$ do not depend on the aperture angle γ of the angular region (figure 1). From (3.12), the eigenvalues $\lambda_{ei}$ of $M_{e}(\gamma ,\eta )$ can be rewritten using the notation (2.29)–(2.30)

3.13 $$\begin{array}{rl} & \lambda_{e1}(\gamma ,\eta )=j(\eta \cos\gamma +\sin\gamma \;\xi_{p}(\eta )),\\ & \lambda_{e2,e3}(\gamma ,\eta )=j(\eta \cos\gamma +\sin\gamma \;\xi_{s}(\eta )),\\ & \lambda_{e4}(\gamma ,\eta )=j(\eta \cos\gamma -\sin\gamma \;\xi_{p}(\eta ))\\ \text{and}\;\; & \lambda_{e5,e6}(\gamma ,\eta )=j(\eta \cos\gamma -\sin\gamma \;\xi_{s}(\eta )), \end{array}\}$$

where the first three $\lambda_{ei}$ are related to progressive waves and the last three to regressive waves according to the definitions reported in §2. The corresponding eigenvectors and reciprocal vectors corresponding to $\lambda_{ei}$ are ${\text{u}}_{i}$ and $\nu_{i}$ reported in (2.32) and (2.33) according to (3.11).

As we will see in the next section, the bi-orthogonal basis ${\text{u}}_{i}$ and $\nu_{i}$ can be used to build the solution of the transverse equations (3.6) in Laplace domain for elastic wave motion problems in an angular region with arbitrary $\alpha_{o}$, i.e. non-planar problems.

## 4. Solution of the oblique equations for angular regions

With reference to figure 1, let us introduce the Laplace transforms of $\psi_{t}(u,v)$ (2.16)

4.1 $${\tilde{\psi }}_{t}(\eta ,v)=\int_{0}^{\infty }e^{j\eta \;u}\psi_{t}(u,v)\;\text{d}u,$$

for regions 1,2 and ${\tilde{\psi }}_{t}(\eta ,v)=\int_{-\infty }^{0}\;e^{j\eta \;u}\psi_{t}(u,v)\;du$ for regions 3,4. The Laplace transforms applied to (3.4) yield

4.2 $$-\frac{\text{d}}{\text{d}v}{\tilde{\psi }}_{t}=M_{e}(\gamma ,\eta )\;{\tilde{\psi }}_{t}+\psi_{s}(v),$$

with

4.3 $$M_{e}(\gamma ,\eta )=M_{eo}-j\eta \;M_{e1}-\eta^{2}M_{e2}.$$

Note that (4.3) and (3.7) share the same symbol and explicit mathematical expression, however, the first is related to a Fourier transform while the second to a Laplace transform, thus obviously they have the same eigenvalues and eigenvectors.

The term $\psi_{s}(v)$ is obtained from the derivative property of the Laplace transform and for each angular region we obtain a different expression. In particular, we indicate with $\psi_{as}(v)$ the value of $\psi_{s}(v)$ on the face a, see figure 1, ($0\leq v<+\infty ,u=0_{+}$), with $\psi_{bs}(v)$ the value of $\psi_{s}(v)$ on the face b ($-\infty \leq v<0,u=0_{+}$), with $\psi_{cs}(v)$ the value of $\psi_{s}(v)$ on the face c ($-\infty \leq v<0,u=0_{-}$) and with $\psi_{ds}(v)$ the value of $\psi_{s}(v)$ on the face d ($0\leq v<+\infty ,u=0_{-}$).

Since (4.2) is a system of six ordinary differential equations of first order with constant coefficients in a semi-infinite interval, we have mainly two methods for its solution: (1) to apply the dyadic Green’s function procedure in v domain, and (2) to apply the Laplace transform in v that yields a linear system of six algebraic equations from which one can write down the general solution in terms of eigenvalues and eigenfunctions. We note that both methods are effective and in particular the second method is more useful for representing the spectral solution in each point of the considered angular region. However, it initially introduces complex functions of two variables. As proposed in the following subsections, we prefer the first method because, by this way, we get the functional equations of the angular regions that involve directly complex functions of one variable.

Using the concept of non-standard Laplace transforms (see §1.4 of [4]), the validity of (4.2) and (4.3) in the absence of sources is extended to the total fields in the presence of plane-wave sources or sources located at infinity from any direction yielding isolated poles in spectral domain.

With reference to figure 1, let us now focus the attention on the angular region 1 in detail. The results for the other regions will follow a similar procedure. We observe that the selection of four angular regions as in figure 1 related to a unique aperture angle γ does not limit the applicability of the method. In fact, all the equations (once derived) can be used with a different appropriate aperture angle just by replacing γ with the proper value. The purpose of deriving the functional equations with a unique γ is related to the fact that we formulate and solve the angular region problems by analysing once and for all the matrix $M_{e}(\gamma ,\eta )$ (4.3). We recall also that the imposition of boundary conditions and media for each region will be made only while examining a practical problem and it yields GWHEs from the functional equations.

### (a) Region 1: u>0,v>0

Focusing the attention on region 1 (figure 1), i.e. u>0,v>0, (4.2) holds with

4.4 $$\psi_{s}(v)=\psi_{as}(v)=-M_{e1}\;\psi_{t}(0_{+},v)+j\eta \;M_{e2}\;\psi_{t}(0_{+},v)-M_{e2}\;\frac{\partial }{\partial u}\psi_{t}(0_{+},v).$$

Equation (4.2) is a system of differential equations of first order of dimension six, whose solution ${\tilde{\psi }}_{t}$ is obtainable as a sum of a particular integral ${\tilde{\psi }}_{p}$ with the general solution of the homogeneous equation ${\tilde{\psi }}_{o}$ [11]

4.5 $${\tilde{\psi }}_{t}={\tilde{\psi }}_{o}+{\tilde{\psi }}_{p}.$$

The solution of the homogeneous equation must satisfy

4.6 $$-\frac{\text{d}}{\text{d}v}{\tilde{\psi }}_{o}=M_{e}(\gamma ,\eta )\;{\tilde{\psi }}_{o}.$$

Considering the solution form ${\tilde{\psi }}_{o}=C\;e_{\;}^{-\lambda (\gamma ,\eta )v}\text{u}(\eta )$, the most general solution is

4.7 $${\tilde{\psi }}_{o}(\gamma ,v)=\sum_{i=1}^{6}C_{i}e^{-\lambda_{ei}(\gamma )\;v}{\text{u}}_{i}(\eta ),$$

where $\lambda_{ei}$ and ${\text{u}}_{i}\;(i=1\cdots 6)$ are the eigenvalues and the eigenvectors of the matrix $M_{e}(\gamma ,\eta )$, respectively, reported at (3.13) and (2.32).

In the presence of a passive medium, following the properties described in §2a, we observe that the first three eigenvalues $\lambda_{ei},\;i=1,2,3$ present non-negative real part and are related to progressive waves along positive v direction while the last three eigenvalues $\lambda_{ei},\;i=4,5,6$ present non-positive real part and are related to regressive waves. The evaluation of the particular integral ${\tilde{\psi }}_{p}(\eta ,v)$ of (4.2) is easier if carried out in dyadic notation, i.e.

4.8 $$-\frac{\text{d}}{\text{d}v}{\tilde{\psi }}_{t}={\underline{M}}_{e}(\gamma ,\eta )\cdot {\tilde{\psi }}_{t}+\psi_{s}(v),$$

where ${\underline{M}}_{e}$ is the dyadic counterpart of the matrix $M_{e}$ assuming canonical basis.^3^ It yields

4.9 $${\tilde{\psi }}_{p}(\eta ,v)=-\int_{0}^{\infty }\underline{G}(v,v^{\prime })\cdot \psi_{s}(v^{\prime })\;\text{d}v^{\prime },$$

where $\underline{G}(v,v^{\prime })$ is the dyadic Green’s function of (4.8), i.e. solution of

4.10 $$\frac{\text{d}}{\text{d}v}\;\underline{G}(v,v^{\prime })+{\underline{M}}_{e}(\gamma ,\eta )\cdot \underline{G}(v,v^{\prime })=\delta (v-v^{\prime }){\underline{1}}_{t}$$

with the unit dyadic ${\underline{1}}_{t}$ of dimension six.

Based on the theory reported in [11,33], we apply the methodology reported in §4 and appendix B of [1], where we build the dyadic Green’s function for arbitrary boundary conditions by selecting progressive and regressive waves in indefinite half-space as homogeneous solutions of (4.10). It yields

4.11 $$\underline{G}(v,v^{\prime })=\left\{ \begin{array}{ll} \sum_{i=1}^{3}{\text{u}}_{i}\nu_{i}\;{\text{e}}^{-\lambda_{ei}(\gamma ,\eta )(v-v^{\prime })}, & v>v^{\prime }\\ -\sum_{i=4}^{6}{\text{u}}_{i}\nu_{i}{\text{e}}^{-\lambda_{ei}(\gamma ,\eta )(v-v^{\prime })}, & v<v^{\prime } \end{array}\right..$$

In our framework, we avoid to impose the boundary condition at this step, since we want to find functional equations that are free of this constraint, as described in [1] based on [11]. Only, while investigating a practical problem, we will impose a boundary condition to the functional equations (for instance in region 1 at face φ=0, i.e. u>0, v=0 and face φ=γ, i.e. u=0, v>0) yielding GWHEs of the problem.

By substituting (4.7) and (4.9) with (4.11) into (4.5), it yields

4.12 $$\begin{array}{rl} {\tilde{\psi }}_{t}(\eta ,v) & \;=\sum_{i=1}^{6}C_{i}{\text{e}}^{-\lambda_{ei}(\gamma )\;v}{\text{u}}_{i}-\sum_{i=1}^{3}{\text{u}}_{i}\nu_{i}\cdot \int_{0}^{v}{\text{e}}^{-\lambda_{ei}(\gamma ,\eta )(v-\;v^{\prime })}\psi_{as}(v^{\prime })\;\text{d}v^{\prime }\\ & \;\;+\sum_{i=4}^{6}{\text{u}}_{i}\nu_{i}\cdot \int_{v}^{\infty }{\text{e}}^{-\lambda_{ei}(\gamma ,\eta )(v-\;v^{\prime })}\psi_{as}(v^{\prime })\;\text{d}v^{\prime }. \end{array}$$

Looking at the asymptotic behaviour of (4.12) for v→+∞ we have that the divergent terms are the ones in $\sum_{i=4}^{6}C_{i}\;e^{-\lambda_{ei}(\gamma )\;v}{\text{u}}_{i}$. For this reason, we assume $C_{i}=0,\;i=4,5,6$. Note in particular the vanishing of the last three integral terms as v→+∞ (last sum in (4.12)).

Setting v=0 in (4.12), we have

4.13 $${\tilde{\psi }}_{t}(\eta ,0)=\sum_{i=1}^{3}C_{i}{\text{u}}_{i}+\sum_{i=4}^{6}{\text{u}}_{i}\nu_{i}\cdot \int_{0}^{\infty }{\text{e}}^{\lambda_{ei}(\gamma ,\eta )v^{\prime }}\psi_{as}(v^{\prime })\;\text{d}v^{\prime }.$$

Multiplying (4.13) by $\nu_{i}(\eta )$ for i=1…6, using bi-orthogonality, we obtain

4.14 $$\begin{array}{rl} & \nu_{i}\cdot {\tilde{\psi }}_{t}(\eta ,0)=C_{i},\;i=1,2,3\\ \text{and}\;\; & \nu_{i}\cdot {\tilde{\psi }}_{t}(\eta ,0)=\nu_{i}\cdot {\overset{⌣}{\psi }}_{as}(-j\lambda_{ei}(\gamma ,\eta )),\;i=4,5,6 \end{array}\},$$

where $\lambda_{ei}(\gamma ,\eta )$ are reported in (3.13) and ${\overset{⌣}{\psi }}_{as}^{\;}(\chi )$ is the Laplace transform in v along face a (v=r in cylindrical coordinates)

4.15 $${\overset{⌣}{\psi }}_{as}(\chi )=\int_{0}^{\infty }\;{\text{e}}^{j\chi v}\psi_{as}(v)\;\text{d}v.$$

We note that in the first three equations of (4.14) we use progressive reciprocal vectors and we obtain $C_{i}$ that are needed in the computation of the homogeneous portion of the solution ${\tilde{\psi }}_{t}(\eta ,v)$ (4.12) through Green’s function method. In particular, the unknowns $C_{i},\;i=1,2,3$ are related to the Laplace transform ${\tilde{\psi }}_{t}(\eta ,0)$ evaluated in the lower face of the angular region (v=0). We now focus attention on the last three equations of (4.14) obtained by using regressive reciprocal vectors that yield the three functional equations of the angular region. We rewrite them as

4.16 $$\nu_{i}\cdot {\tilde{\psi }}_{t}(\eta ,0)=\nu_{i}\cdot {\overset{⌣}{\psi }}_{as}(-m_{ai}(\gamma ,\eta )),\;i=4,5,6,$$

with

4.17 $$\begin{array}{rl} & m_{a4}(\gamma ,\eta )=m_{p}(\gamma ,\eta )=j\lambda_{e4}(\gamma ,\eta )=-\eta \cos\gamma +\xi_{p}\sin\gamma \\ \text{and}\;\; & m_{a5,a6}(\gamma ,\eta )=m_{s}(\gamma ,\eta )=j\lambda_{e5,e6}(\gamma ,\eta )=-\eta \cos\gamma +\xi_{s}\sin\gamma . \end{array}\}$$

In (4.16), the Laplace transforms of combinations of the field components defined on the boundaries of an angular region, i.e. v=0 (face o) and u=0 (face a) in figure 1, are related to each other. These functional equations are the starting point to define the GWHEs of region 1. They are valid for any linear isotropic elastic medium filling the region. Moreover, in (4.16), we note that the reciprocal vectors and eigenvectors do not appear in the definitions of the Laplace transforms on the field. Only the eigenvalues are used as an argument of the Laplace transforms on the right-hand side. In the following, we apply the notation + to ${\tilde{\psi }}_{t}(\eta ,0)$ and ${\overset{⌣}{\psi }}_{as}(-m_{ai}(\gamma ,\eta ))$, i.e. ${\tilde{\psi }}_{t+}(\eta ,0)$ and ${\overset{⌣}{\psi }}_{as+}(-m_{ai}(\gamma ,\eta ))$, to highlight that these Laplace transforms are plus functions respectively in η and $\chi =-m_{ai}(\gamma ,\eta )$, i.e. they are regular in the upper half plane of the complex plane η and χ.

Note that the functional equations (4.16) contain spectral unknowns defined into two different complex planes (η and $\chi =-m_{ai}(\gamma ,\eta ))$ related together via (4.17) and thus, when we impose the boundary conditions we get GWHEs and not CWHEs (except in the case of γ=π).

Explicit forms of functional equations (4.16) are obtained and reported in §5 for isotropic media, however the theory reported in this paper can be applied to more complex media.

### (b) From region 1 to the other angular regions

Now, let us repeat the procedure for region 2 (figure 1), i.e. u>0,v<0. The solution ${\tilde{\psi }}_{t}(\eta ,v)$ of the system of differential equations of first order of dimension six (4.2) is obtainable as sum (4.5) of the general homogeneous solution ${\tilde{\psi }}_{o}$ with a particular integral ${\tilde{\psi }}_{p}$ defined in terms of

4.18 $$\psi_{s}(v)=\psi_{bs}(v)=-M_{e1}\;\psi_{t}(0_{+},v)+j\eta M_{e2}\;\psi_{t}(0_{+},v)-M_{e2}\;\frac{\partial }{\partial u}\psi_{t}(0_{+},v)$$

in region 2 (v<0). We note that (4.18) is equal to (4.4) but with different support in v. The homogeneous solution takes the form (4.7). In the presence of a passive medium, we recall that the first three eigenvalues present non-negative real part and are related to progressive waves along positive v while the last three eigenvalues present non-positive real part and are related to regressive waves, thus looking at the asymptotic behaviour of (4.7) for v→−∞ we have $C_{i}=0,\;i=1,2,3$. Once the dyadic Green’s function specialized for region 2 is obtained, the solution is

4.19 $$\begin{array}{rl} {\tilde{\psi }}_{t}(\eta ,v) & \;=\sum_{i=4}^{6}C_{i}{\text{u}}_{i}{\text{e}}^{-\lambda_{ei}(\gamma ,\eta )\;v}-\sum_{i=1}^{3}{\text{u}}_{i}\nu_{i}\cdot \int_{-\infty }^{v}{\text{e}}^{-\lambda_{ei}(\gamma ,\eta )(v-\;v^{\prime })}\psi_{bs}(v^{\prime })dv^{\prime }\\ & \;+\;\sum_{i=4}^{6}{\text{u}}_{i}\nu_{i}\cdot \int_{v}^{0}{\text{e}}^{-\lambda_{ei}(\gamma ,\eta )(v-\;v^{\prime })}\psi_{bs}(v^{\prime })\;\text{d}v^{\prime } \end{array}$$

before imposing the boundary conditions. Setting v=0 in (4.19), we have

4.20 $${\tilde{\psi }}_{t}(\eta ,0)=\sum_{i=4}^{6}C_{i}{\text{u}}_{i}-\sum_{i=1}^{3}{\text{u}}_{i}\nu_{i}\cdot \int_{-\infty }^{0}\;{\text{e}}^{\lambda_{ei}(\gamma ,\eta )v^{\prime }}\psi_{bs}(v^{\prime })\;\text{d}v^{\prime }.$$

Multiplying (4.20) by $\nu_{i}(\eta )$ for i=1…6, using bi-orthogonality, we obtain

4.21 $$\begin{array}{rl} & \nu_{i}\cdot {\tilde{\psi }}_{t}(\eta ,0)=C_{i},\;i=4,5,6\\ \text{and}\;\; & \nu_{i}\cdot {\tilde{\psi }}_{t}(\eta ,0)=-\nu_{i}\cdot {\overset{⌣}{\psi }}_{bs}(j\lambda_{ei}(\gamma ,\eta )),\;i=1,2,3 \end{array}\}$$

where $\lambda_{ei}(\gamma ,\eta )$ are reported in (3.13) and where

4.22 $${\overset{⌣}{\psi }}_{bs}(\chi )=\int_{-\infty }^{0}\;{\text{e}}^{-j\chi v}\psi_{bs}(v)\;\text{d}v=\int_{0}^{\infty }\;{\text{e}}^{j\chi r}\psi_{bs}(-r)\;\text{d}r,$$

is the left Laplace transform of $\psi_{bs}(v)$ in v along face b (figure 1) or the Laplace transform in r of $\psi_{bs}(-r)$ in cylindrical coordinates (r,φ,z). The properties of (4.21) are the same as for region 1. In particular, we focus attention on the last three equations obtained by using progressive reciprocal vectors that yield the functional equations of the angular region. We rewrite them as

4.23 $$\nu_{i}\cdot {\tilde{\psi }}_{t}(\eta ,0)=-\nu_{i}\cdot {\overset{⌣}{\psi }}_{bs}(-m_{bi}(\gamma ,\eta )),\;i=1,2,3$$

with

4.24 $$\begin{array}{rl} & m_{b1}(\gamma ,\eta )=m_{pb}(\gamma ,\eta )=-j\lambda_{e1}(\gamma ,\eta )=\eta \cos\gamma +\xi_{p}\sin\gamma ,\\ \text{and}\;\; & m_{b2,b3}(\gamma ,\eta )=m_{sb}(\gamma ,\eta )=-j\lambda_{e2,e3}(\gamma ,\eta )=\eta \cos\gamma +\xi_{s}\sin\gamma . \end{array}\}$$

In (4.23), the Laplace transforms of combinations of the field components defined on the boundaries of an angular region, i.e. v=0 (face o) and u=0 (face b) in figure 1, are related together. These functional equations are the starting point to define the GWHEs of region 2 by imposing boundary conditions and in particular they can be coupled to the ones of region 1 to build a structure with two angular regions with different elastic properties.

Observing (4.23), we note that at the second members we have that, in general, ${\overset{⌣}{\psi }}_{bs}(-m_{bi}(\gamma ,\eta ))$ contains discontinuous field components at the boundary u=0,v<0 of the angular region, while ${\tilde{\psi }}_{t}(\eta ,0)$ (by definition 2.16) is continuous at the boundary u>0,v=0.

Similarly to what has been done in [1] for electromagnetic applications, we can repeat the procedure to obtain functional equations for regions 3 and 4 (figure 1).

## 5. Explicit form of the functional equations for non-planar (three-dimensional) problems in angular regions

In this section, according to our opinion, we deduce and report for the first time in the literature explicit spectral functional equations in algebraic form for the non-planar (three-dimensional) elastic scattering problem in isotropic angular regions with arbitrary boundary conditions.

### (a) Explicit form for region 1

We remark that (4.16) are the functional equations of region 1 for an elastic wave motion problem in an isotropic medium at skew (non-planar) incidence ($\alpha_{o}\neq 0$). The functional equations for the two-dimensional (planar and anti-planar) problems are a particular case of the general wave motion problem with $\alpha_{o}=0$. In the following, we demonstrate for validation that the GWHEs obtained from the proposed functional equations enforcing the boundary conditions and the functional equations obtained in [14] using the Gautesen (Kirchhoff) integral representations in the natural domain are identical, although the applied notations are different from each other and not immediate in the comparison.

To explicitly represent (4.16) in region 1, we need $\nu_{i}$ reported in the rows of V (2.33), the Laplace transform of the field ${\tilde{\psi }}_{t}(\eta ,0)$ along $x,u>0,v=0_{+}$ (face o, see figure 1) and the Laplace transform ${\overset{⌣}{\psi }}_{as}(-m_{ai}(\gamma ,\eta ))$ along $x,u=0_{+},v>0$ (face a, see figure 1). An important property of functional equations is that they report combinations of field components that are continuous on the two boundaries of the angular region. This property is fundamental to enforce boundary conditions in particular while connecting the angular region to a different body. We observe that, while ${\tilde{\psi }}_{t}(\eta ,0)$ is continuous at face o by definition (2.16), we need some mathematical manipulations to demonstrate that ${\overset{⌣}{\psi }}_{as}(-m_{ai}(\gamma ,\eta ))$ (4.4) is defined in terms of continuous field components at face a for an arbitrary aperture angle γ, since its expression contains potential discontinuous components such as derivatives of the field. The proof follows. According to a local-to-face-a Cartesian coordinate system X,Y,Z≡z (figure 1), we have that the continuous components of the field are $T_{YY},T_{YZ},T_{XY},v_{X},v_{Y},v_{Z}$, but ${\overset{⌣}{\psi }}_{as}(-m_{ai}(\gamma ,\eta ))$ and thus $\psi_{s}(v)=\psi_{as}(v)$ are originally defined in terms of $T_{yy},T_{yz},T_{xy},v_{x},v_{y},v_{z}$ and their derivatives, which in general are discontinuous, see (4.15), (4.4) and (2.16). In fact, the explicit form of $\psi_{as}(v)$ (4.4), using (3.5) and (2.23)–(2.25), is:

5.1 $$\begin{array}{rl} & \psi_{as}(v)\\ & \;\;=\left(\begin{array}{c} T_{yy}\cos(\gamma )-T_{xy}\sin(\gamma )\\ \frac{{k_{s}}^{3}T_{yz}\cos(\gamma )+Z_{o}\sin(\gamma )(jD_{u}v_{z}{k_{s}}^{2}-4\alpha_{o}{k_{p}}^{2}v_{x}+{k_{s}}^{2}(\eta v_{z}+3\alpha_{o}v_{x}))}{{k_{s}}^{3}}\\ \frac{{k_{s}}^{3}T_{xy}\cos(\gamma )+\sin(\gamma )(2{k_{p}}^{2}(-2jD_{u}v_{x}Z_{o}+k_{s}T_{yy}-2Z_{o}(\alpha_{o}v_{z}+\eta v_{x}))+{k_{s}}^{2}(-k_{s}T_{yy}+Z_{o}(4jD_{u}v_{x}+3\alpha_{o}v_{z}+4\eta v_{x})))}{{k_{s}}^{3}}\\ v_{x}\cos(\gamma )-v_{y}\sin(\gamma )\\ v_{x}\sin(\gamma )\left(\frac{2{k_{p}}^{2}}{{k_{s}}^{2}}-1\right)+v_{y}\cos(\gamma )\\ v_{z}\cos(\gamma ) \end{array}\right) \end{array}$$

with $D_{u}=\partial /\partial u{|}_{u=0+}$. As a first step to check the properties of (5.1) on face a, we derive expressions for $D_{u}$ components of the velocity that appears at the second and third components of (5.1). Noting that $D_{u}=D_{x}$ and $D_{z}=-j\alpha_{o}$, from the fourth and the eighth basic equations reported in (2.15), we have

5.2 $$\begin{array}{rl} & D_{u}v_{x}=\frac{jk_{s}[2k_{p}^{2}(T_{xx}-T_{yy}-T_{zz})+k_{s}^{2}(-2T_{xx}+T_{yy}+T_{zz})]}{8k_{p}^{2}Z_{o}-6k_{s}^{2}Z_{o}}\\ \text{and}\;\; & D_{u}v_{z}=\frac{jk_{s}^{\;}T_{xz}}{Z_{o}}+j\alpha_{o}v_{x}. \end{array}\}$$

Substituting (5.2) into (5.1), we get an expression of $\psi_{as}(v)$ in terms of T and v components without derivatives but still defined in terms of x,y,z. Now, in order to rewrite $\psi_{s}(v)=\psi_{as}(v)=\psi_{s}(X,Y=0)$ only in terms of the local continuous components $T_{YY},T_{YZ},T_{XY},v_{X},v_{Y},v_{Z}$ (face a, see figure 1), we formulate the rotational problem between components along x,y,z with respect to their definition along X,Y,Z. Without loss of generality, assuming 0<γ<π,

5.3 $$\begin{array}{rl} T & \;=R_{a}^{-1}\;T_{a}\;R_{a}, \end{array}$$

5.4 $$\begin{array}{rl} T & \;=\left(\begin{array}{ccc} T_{xx} & T_{xy} & T_{xz}\\ T_{xy} & T_{yy} & T_{yz}\\ T_{xz} & T_{yz} & T_{zz} \end{array}\right),\;T_{a}=\left(\begin{array}{ccc} T_{XX} & T_{XY} & T_{XZ}\\ T_{XY} & T_{YY} & T_{YZ}\\ T_{XZ} & T_{YZ} & T_{ZZ} \end{array}\right),\\ R_{a} & \;=\left(\begin{array}{ccc} \cos(\gamma ) & \sin(\gamma ) & 0\\ -\sin(\gamma ) & \cos(\gamma ) & 0\\ 0 & 0 & 1 \end{array}\right), \end{array}$$

5.5 $$\begin{array}{rl} \text{and}\;\;\text{v} & \;=R_{a}^{-1}\;{\text{v}}_{a},\;\text{v}=\left(\begin{array}{c} v_{x}\\ v_{y}\\ v_{z} \end{array}\right),\;{\text{v}}_{a}=\left(\begin{array}{c} v_{X}\\ v_{Y}\\ v_{Z} \end{array}\right). \end{array}$$

Substituting (5.3) and (5.5) into (5.1) after the application of (5.2), it yields an expression of $\psi_{as}(v)$ in terms of the components $T_{a}$ and $v_{a}$ in X, Y, Z

5.6 $$\begin{array}{rl} & \psi_{as}(v)\\ & \;\;=\left(\begin{array}{c} T_{XY}\sin(\gamma )+T_{YY}\cos(\gamma )\\ \frac{\alpha_{o}Z_{o}({k_{s}}^{2}-2{k_{p}}^{2})(v_{X}\sin(2\gamma )+v_{Y}\cos(2\gamma ))}{{k_{s}}^{3}}-\frac{\alpha_{o}v_{Y}Z_{o}({k_{s}}^{2}-2{k_{p}}^{2})}{{k_{s}}^{3}}+\frac{\eta v_{Z}Z_{o}\sin(\gamma )}{k_{s}}+T_{YZ}\\ \psi_{as3}(v)\\ v_{X}\cos(2\gamma )-v_{Y}\sin(2\gamma )\\ \frac{v_{Y}({k_{p}}^{2}\cos(2\gamma )-{k_{p}}^{2}+{k_{s}}^{2})+{k_{p}}^{2}v_{X}\sin(2\gamma )}{{k_{s}}^{2}}\\ v_{Z}\cos(\gamma ) \end{array}\right), \end{array}$$

where

5.7 $$\begin{array}{rl} \psi_{as3}(v)(4{k_{p}}^{2}{k_{s}}^{3}-3{k_{s}}^{5}) & \;={k_{s}}^{3}T_{XY}\cos(\gamma )(4{k_{p}}^{2}-3{k_{s}}^{2})+\sin(\gamma )[\alpha_{o}(-v_{Z})Z_{o}{\left(4{k_{p}}^{2}-3{k_{s}}^{2}\right)}^{2}\\ & \;\;+k_{s}(4{k_{p}}^{4}(T_{XX}+T_{YY}-T_{ZZ})-2{k_{p}}^{2}{k_{s}}^{2}(2T_{XX}+4T_{YY}-3T_{ZZ})\\ & \;\;+{k_{s}}^{4}(T_{XX}+4T_{YY}-2T_{ZZ}))]\\ & \;\;+4\eta Z_{o}(4{k_{p}}^{4}-7{k_{p}}^{2}{k_{s}}^{2}+3{k_{s}}^{4})(v_{Y}\sin(\gamma )-v_{X}\cos(\gamma )). \end{array}$$

We recall that the procedure aims at finding $\psi_{as}(v)$ in terms of the continuous field $T_{YY}$, $T_{YZ}$, $T_{XY}$, $v_{X}$, $v_{Y}$, $v_{Z}$. The result of the proposed substitutions is that the components of $\psi_{as}(v)$ (5.6) are all expressed in terms of the continuous field except the component 3. In fact, from the beginning, the component 3 of (5.1) contains $D_{u}v_{x}$ that is represented by the first of (5.2) where the discontinuous $T_{xx},T_{zz}$ are present. The subsequent application of (5.3) and (5.5) does not change the properties $\psi_{as}(v)$ in terms of continuous components and in particular the third component contains the discontinuous components $T_{XX},T_{ZZ}$ as reported in (5.6) with (5.7). Noting that the basic equations (2.15) are invariant for rotations of the coordinate axes, by applying the sixth of (2.15) in X,Y,Z coordinates we get

5.8 $$T_{ZZ}=\frac{k_{s}({k_{s}}^{2}-2{k_{p}}^{2})(T_{XX}+T_{YY})+2\alpha_{o}v_{Z}Z_{o}(4{k_{p}}^{2}-3{k_{s}}^{2})}{2({k_{s}}^{3}-{k_{p}}^{2}k_{s})}.$$

The substitution of (5.8) into $\psi_{as3}(v)$ (5.7), after mathematical manipulations, yields an expression in terms of continuous field, whose embedding in (5.6) gives a representation of $\psi_{as}(v)$ only in terms of continuous field at face a

5.9 $$\begin{array}{rl} & \psi_{as}(v)\\ & \;\;=\left(\begin{array}{c} T_{XY}\sin(\gamma )+T_{YY}\cos(\gamma )\\ \frac{\alpha_{o}Z_{o}({k_{s}}^{2}-2{k_{p}}^{2})(v_{X}\sin(2\gamma )+v_{Y}\cos(2\gamma ))}{{k_{s}}^{3}}-\frac{\alpha_{o}v_{Y}Z_{o}({k_{s}}^{2}-2{k_{p}}^{2})}{{k_{s}}^{3}}+\frac{\eta v_{Z}Z_{o}\sin(\gamma )}{k_{s}}+T_{YZ}\\ \frac{\sin(\gamma )(4\eta v_{Y}Z_{o}\sin(\gamma )(k_{p}^{2}-k_{s}^{2})+{k_{s}}^{2}(\alpha_{o}v_{Z}Z_{o}-k_{s}T_{YY}))+2\eta v_{X}Z_{o}\sin(2\gamma )(k_{s}-k_{p})(k_{p}+k_{s})+{k_{s}}^{3}T_{XY}\cos(\gamma )}{{k_{s}}^{3}}\\ v_{X}\cos(2\gamma )-v_{Y}\sin(2\gamma )\\ \frac{v_{Y}({k_{p}}^{2}\cos(2\gamma )-{k_{p}}^{2}+{k_{s}}^{2})+{k_{p}}^{2}v_{X}\sin(2\gamma )}{{k_{s}}^{2}}\\ v_{Z}\cos(\gamma ) \end{array}\right). \end{array}$$

From (5.9), we note that $\psi_{as}(v)$ is defined only in terms of continuous field component at face a. Now, the application of Laplace transform (4.15) to $\psi_{as}(v)$ yields the explicit expression of the spectral functional equations (4.16) for region 1 in terms of continuous components. We remark that this property is fundamental to easily impose impenetrable boundary conditions and to couple region 1 with other penetrable surrounding regions of arbitrary geometry and in general non-homogeneous to region 1.

The property of the elastic wave motion problem to be formulated in terms of a differential problem (4.2) with sources $\psi_{as}(v)$ (5.9) defined only in term of continuous field on the boundary represents an *equivalence theorem in elasticity* analogous to the well-known equivalence theorem in electromagnetism. In fact, the solution is given by ${\tilde{\psi }}_{t}(\eta ,v)$ (4.12) through Green’s function formulation only in terms of continuous components on the two faces of the angular region ($C_{i}$ on face o and $\psi_{as}(v)$ on face a), see (4.12)–(4.14). This property is corresponding to the well-known Schelkunoff’s equivalence theorem together with the uniqueness theorem in electromagnetics [34], where the equivalent sources are defined in terms of the components of electromagnetic field E,H tangent (continuous) to (at) the boundaries. A tentative text may be the following.

*Equivalence theorem in elasticity:* *A field in a lossy region is uniquely specified by the sources within the region plus the continuous components of the fields over the boundary.*

In order to avoid trivial identities for $\alpha_{o}=0$ and in order to simplify a little the explicit form of functional equations (4.16), we redefine the reciprocal vectors $\nu_{i}$ starting from the rows V(i,:), i=1..6 of (2.33) according to the following scaling (reciprocal vectors as eigenvectors are defined up to a multiplicative constant):

5.10 $$\begin{array}{rl} & \nu_{1}=\frac{2Z_{o}\xi_{p}k_{s}^{2}V(1,:)}{\alpha_{o}},\;\nu_{2}=2Z_{o}\xi_{s}k_{s}^{2}V(2,:),\;\nu_{3}=2Z_{o}\xi_{s}k_{s}^{2}V(3,:)\\ & \nu_{4}=\frac{2Z_{o}\xi_{p}k_{s}^{2}V(4,:)}{\alpha_{o}},\;\nu_{5}=2Z_{o}\xi_{s}k_{s}^{2}V(5,:),\;\nu_{6}=2Z_{o}\xi_{s}k_{s}^{2}V(6,:). \end{array}\}$$

With (5.10), (4.16) take the form (5.11)–(5.13), where the T,v quantities with lower-case subscripts in the LHS of the equations are defined for $u>0,\;v=0_{+}$ and are Laplace transforms in η, while the T,v quantities with upper-case subscripts are defined for $u=0_{+},\;v>0$ and are Laplace transforms in $-m_{p}$, $-m_{s}$, $-m_{s}$, respectively, in the RHS of (5.11), (5.12), (5.13).

5.11 $$\begin{array}{rl} & k_{s}(-T_{yy}\xi_{p}+\eta T_{xy}+\alpha_{o}T_{yz})+Z_{o}[2\xi_{p}(\eta v_{x}+\alpha_{o}v_{z})+v_{y}({\alpha_{o}}^{2}+\eta^{2}-\xi_{s}^{2})]\\ & \;=Z_{o}[v_{Y}({\alpha_{o}}^{2}+{k_{p}}^{2}-{k_{s}}^{2})+v_{X}\sin(2\gamma )(\eta^{2}-\xi_{p}^{2})+2\xi_{p}(\eta v_{X}\cos(2\gamma )\\ & \;\;-\eta v_{Y}\sin(2\gamma )+\alpha_{o}v_{Z}\cos(\gamma ))+v_{Y}\cos(2\gamma )(\eta^{2}-\xi_{p}^{2})+2\alpha_{o}\eta v_{Z}\sin(\gamma )]\\ & \;\;+k_{s}[-\xi_{p}(T_{XY}\sin(\gamma )+T_{YY}\cos(\gamma ))+\eta T_{XY}\cos(\gamma )-\eta T_{YY}\sin(\gamma )+\alpha_{o}T_{YZ}], \end{array}$$

5.12 $$\begin{array}{rl} & k_{s}\xi_{s}(\eta T_{xy}+\alpha_{o}T_{yz})+k_{s}T_{yy}({\alpha_{o}}^{2}+\eta^{2})\\ & \;\;+Z_{o}[\xi_{s}^{2}(\eta v_{x}+\alpha_{o}v_{z})+2v_{y}({\alpha_{o}}^{2}+\eta^{2})\xi_{s}-({\alpha_{o}}^{2}+\eta^{2})(\eta v_{x}+\alpha_{o}v_{z})]\\ & \;=k_{s}\xi_{s}[\eta T_{XY}\cos(\gamma )-\eta T_{YY}\sin(\gamma )+\alpha_{o}T_{YZ}]\\ & \;\;+k_{s}({\alpha_{o}}^{2}+\eta^{2})[T_{XY}\sin(\gamma )+T_{YY}\cos(\gamma )]\\ & \;\;+Z_{o}\{\xi_{s}[\xi_{s}(\eta v_{X}\cos(2\gamma )-\eta v_{Y}\sin(2\gamma )+\alpha_{o}v_{Z}\cos(\gamma ))+v_{X}({\alpha_{o}}^{2}+2\eta^{2})\sin(2\gamma )\\ & \;\;+v_{Y}({\alpha_{o}}^{2}+2\eta^{2})\cos(2\gamma )+{\alpha_{o}}^{2}v_{Y}+2\alpha_{o}\eta v_{Z}\sin(\gamma )]\\ & \;\;-({\alpha_{o}}^{2}+\eta^{2})[\eta v_{X}\cos(2\gamma )-\eta v_{Y}\sin(2\gamma )+\alpha_{o}v_{Z}\cos(\gamma )]\}, \end{array}$$

5.13 $$\begin{array}{rl} & {k_{s}}^{3}T_{yz}+\xi_{s}\{Z_{o}[{k_{s}}^{2}v_{z}+2\alpha_{o}v_{y}\xi_{s}-2\alpha_{o}(\eta v_{x}+\alpha_{o}v_{z})]+\alpha_{o}k_{s}T_{yy}\}-\alpha_{o}k_{s}(\eta T_{xy}+\alpha_{o}T_{yz})\\ & \;=Z_{o}\{\alpha_{o}\sin(2\gamma )[v_{X}(-{\alpha_{o}}^{2}-2\eta^{2}+{k_{s}}^{2})+2\eta v_{Y}\xi_{s}]-\alpha_{o}\cos(2\gamma )[v_{Y}({\alpha_{o}}^{2}+2\eta^{2}-{k_{s}}^{2})\\ & \;\;+2\eta v_{X}\xi_{s}]+v_{Z}\cos(\gamma )({k_{s}}^{2}-2{\alpha_{o}}^{2})\xi_{s}+\eta v_{Z}\sin(\gamma )({k_{s}}^{2}-2{\alpha_{o}}^{2})+\alpha_{o}v_{Y}({k_{s}}^{2}-{\alpha_{o}}^{2})\}\\ & \;\;+k_{s}\{T_{YZ}({k_{s}}^{2}-{\alpha_{o}}^{2})+\alpha_{o}\xi_{s}[T_{XY}\sin(\gamma )+T_{YY}\cos(\gamma )]+\alpha_{o}\eta [T_{YY}\sin(\gamma )-T_{XY}\cos(\gamma )]\}. \end{array}$$

We remark that (5.11)–(5.13) are the functional equations of region 1 for an elastic wave motion problem in an isotropic medium at skew (non-planar) incidence ($\alpha_{o}\neq 0$). These equations, according to our opinion, are deduced and reported for the first time in the literature.

In particular, by applying the traction-free boundary conditions ($T_{xy}=T_{yy}=T_{yz}=T_{XY}=T_{YY}=T_{YZ}=0$), (5.11)–(5.13) becomes GWHEs formulating the three-dimensional elastic wedge problem considered in [17]. This formulation is important because it allows to get semianalytical solutions via the Fredholm factorization method as developed by the authors in [4]. According to the authors’ opinion, this technique constitutes a very power tool for the accurate approximate solutions of arbitrary WH equations. We remark that the GWHEs are algebraic, while in [17], the solution is obtained by functional equations written in terms of singular integral operators and solved by numerical technique. We assert that the semianalytic solution using the Fredholm factorization method allows physical insights by asymptotics in spectral domain.

### (b) Explicit form for region 2

**Figure 2.**
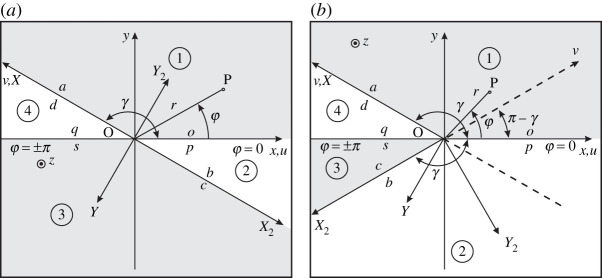
Angular regions and oblique Cartesian coordinates. (*a*) re-reports figure 1 for convenience and it is the reference for the theory developed in the previous sections. (*b*) shows the new framework of the space divided into four angular regions for wedge structures. We note symmetry between regions 1(3) and 2(4). The figure reports the x,y,z Cartesian coordinates and r, φ, z cylindrical coordinates useful to define the oblique Cartesian coordinate system u, v, z with reference to the angular region 1 0<φ<γ with 0<γ<π and u, v, z with reference to the angular region 2 (only in (*b*)). The face boundaries are labelled a, b, c, d, o, p, q, s. The figure reports also the local-to-face-a Cartesian coordinate system X,Y,Z≡z and the local-to-face-b Cartesian coordinate system $X_{2},Y_{2},Z_{2}\equiv z$ (only in (*b*)). The X,Y,Z≡z and $X_{2},Y_{2},Z_{2}\equiv z$ Cartesian coordinate systems are obtained from x,y,z Cartesian coordinate system by rotation, respectively, for a positive γ and −γ.

In this subsection, we repeat the procedure reported in §5a for region 2 (figure 1), i.e. u>0, v<0, but with a different aperture angle, as reported in figure 2*b*: the aperture angle of region 2 is γ instead of π−γ as originally taken in figure 1. This difference is of great utility in the analysis of wedge structures with symmetries. For this purpose, we first start by deriving functional equations of region 2 (4.23) with the original aperture angle γ (figures 1 and 2*a*) for an elastic wave motion problem in an isotropic medium at skew (non-planar) incidence ($\alpha_{o}\neq 0$). Second, we apply the change in the aperture angle and the rotation of the local reference system. To explicitly represent (4.23) for region 2, we need $\nu_{i}$ reported in the rows of V (2.33), the Laplace transform ${\tilde{\psi }}_{t}(\eta ,0)$ along x,u>0,v=0 (face o) and the Laplace transform ${\overset{⌣}{\psi }}_{bs}(-m_{bi}(\gamma ,\eta ))$ along x,u=0,v<0 (face b). We observe that, while ${\tilde{\psi }}_{t}(\eta ,0)$ is continuous at face p by definition (2.16), we need some mathematical manipulations to demonstrate that ${\overset{⌣}{\psi }}_{bs}(-m_{bi}(\gamma ,\eta ))$ (4.18) is defined in terms of continuous field components at face b for an arbitrary aperture angle γ, since its expression contains potential discontinuous components such as derivatives of the field.

According to a local-to-face-b Cartesian coordinate system $X_{2},Y_{2},Z_{2}\equiv z$ (figure 2), we have that the continuous components of the field are $T_{Y2Y2}$, $T_{Y2Z2}$, $T_{X2Y2}$, $v_{X2}$, $v_{Y2}$, $v_{Z2}$, but ${\overset{⌣}{\psi }}_{bs}(-m_{bi}(\gamma ,\eta ))$ and thus $\psi_{s}(v)=\psi_{bs}(v)$ are defined in terms of $T_{yy},T_{yz},T_{xy},v_{x},v_{y},v_{z}$ and their derivatives, which in general are discontinuous, see (4.22), (4.18) and (2.16). In fact, the explicit form of $\psi_{bs}(v)$ (4.18), using (3.5) and (2.23)–(2.25), yields the same expression of $\psi_{as}(v)$ given in (5.1), even if $\psi_{bs}(v)$ is defined for v<0 and $\psi_{as}(v)$ for v>0. Following the steps done for $\psi_{as}(v)$ in region 1, we derive expressions for $D_{u}$ components of the velocity appearing in (5.1). Noting that $D_{u}=D_{x}$ and $D_{z}=-j\alpha_{o}$, from the fourth and the eighth basic equations reported in (2.15), we have (5.2) that substituted into $\psi_{bs}(v)$ yields an expression in terms of T and v components without derivatives but still defined in terms of the coordinate system x,y,z.

Now, in order to rewrite $\psi_{s}(v)=\psi_{bs}(v)=\psi_{s}(X_{2},Y_{2}=0)$ only in terms of the local continuous components $T_{Y2Y2}$, $T_{Y2Z2},T_{X2Y2}$, $v_{X2}$, $v_{Y2}$, $v_{Z2}$ (face b), we formulate the rotational problem between components along x, y, z with respect to their definition along $X_{2}$, $Y_{2}$, $Z_{2}$. The required rotation in figure 2*a* is −π+γ. Now, let us introduce also the change of aperture angle from γ to π−γ as in figure 2*b*. This change of aperture angle impacts on the definitions of $M_{ei}$ matrices (due to the replacement of γ with π−γ) and then $\psi_{bs}(v)$ that now becomes different from $\psi_{as}(v)$. In the new region 2 (figure 2*b*), the rotation relations (5.3)–(5.5) of region 1 are replaced by the relations for region 2 where we have performed the substitution γ→−π+γ (rotation) and γ→π−γ (change of aperture angle), thus γ→−γ. It yields:

5.14 $$\begin{array}{rl} T & \;={R_{b}}^{-1}\;T_{b}\;R_{b}, \end{array}$$

5.15 $$\begin{array}{rl} T & \;=\left(\begin{array}{ccc} T_{xx} & T_{xy} & T_{xz}\\ T_{xy} & T_{yy} & T_{yz}\\ T_{xz} & T_{yz} & T_{zz} \end{array}\right),\;T_{b}=\left(\begin{array}{ccc} T_{X2X2} & T_{X2Y2} & T_{X2Z2}\\ T_{X2Y2} & T_{Y2Y2} & T_{Y2Z2}\\ T_{X2Z2} & T_{Y2Z2} & T_{Z2Z2} \end{array}\right),\\ R_{b} & \;=\left(\begin{array}{ccc} \cos(\gamma ) & -\sin(\gamma ) & 0\\ \sin(\gamma ) & \cos(\gamma ) & 0\\ 0 & 0 & 1 \end{array}\right), \end{array}$$

5.16 $$\begin{array}{rl} \text{and}\;\;\text{v} & \;={R_{b}}^{-1}\;{\text{v}}_{b},\;\text{v}=\left(\begin{array}{c} v_{x}\\ v_{y}\\ v_{z} \end{array}\right),\;{\text{v}}_{b}=\left(\begin{array}{c} v_{X2}\\ v_{Y2}\\ v_{Z2} \end{array}\right). \end{array}$$

Substituting (5.14) and (5.16) into $\psi_{bs}(v)$ (same expression of $\psi_{as}(v)$ (5.1)) after the application of (5.2) and (5.8) in $X_{2}$, $Y_{2}$, $Z_{2}$ coordinates, it yields an expression of $\psi_{bs}(v)$ in terms of the continuous (at face b) components $T_{Y2Y2}$, $T_{Y2Z2}$, $T_{X2Y2}$, $v_{X2}$, $v_{Y2}$, $v_{Z2}$:

5.17 $$\begin{array}{rl} & \psi_{bs}(v)\\ & \;\;=\left(\begin{array}{c} T_{X_{2}Y_{2}}\sin(\gamma )-T_{Y_{2}Y_{2}}\cos(\gamma )\\ \frac{\alpha_{o}v_{X_{2}}Z_{o}\sin(2\gamma )({k_{s}}^{2}-2{k_{p}}^{2})+\alpha_{o}v_{Y_{2}}Z_{o}\cos(2\gamma )(2{k_{p}}^{2}-{k_{s}}^{2})+\alpha_{o}v_{Y_{2}}Z_{o}({k_{s}}^{2}-2{k_{p}}^{2})+\eta {k_{s}}^{2}v_{Z_{2}}Z_{o}\sin(\gamma )}{{k_{s}}^{3}}-T_{Y_{2}Z_{2}}\\ \frac{\sin(\gamma )[4\eta v_{Y_{2}}Z_{o}\sin(\gamma )({k_{s}}^{2}-{k_{p}}^{2})+{k_{s}}^{2}(\alpha_{o}v_{Z_{2}}Z_{o}-k_{s}T_{Y_{2}Y_{2}})]+2\eta v_{X_{2}}Z_{o}\sin(2\gamma )({k_{s}}^{2}-{k_{p}}^{2})-{k_{s}}^{3}T_{X_{2}Y_{2}}\cos(\gamma )}{{k_{s}}^{3}}\\ -v_{X_{2}}\cos(2\gamma )-v_{Y_{2}}\sin(2\gamma )\\ \frac{{k_{p}}^{2}[v_{X_{2}}\sin(2\gamma )-v_{Y_{2}}\cos(2\gamma )]+v_{Y_{2}}({k_{p}}^{2}-{k_{s}}^{2})}{{k_{s}}^{2}}\\ -v_{Z_{2}}\cos(\gamma ) \end{array}\right). \end{array}$$

Now, the application of Laplace transform (4.22) to $\psi_{bs}(v)$ yields the explicit expression of the spectral functional equations (4.16) for region 2 in terms of continuous components.

Again the property of the elastic wave motion problem to be formulated in terms of a differential problem (4.2) with sources $\psi_{bs}(v)$ (5.17) defined only in terms of continuous field on the boundary represents an *equivalence theorem in elasticity* for region 2, as discussed in §5a.

As done for region 1, in order to avoid trivial identities for $\alpha_{o}=0$ and in order to simplify a little the explicit form of (4.23), we redefine the reciprocal vectors as reported in (5.10). With (5.10), (4.23) take the form (5.18)–(5.20), where the T,v quantities with lower-case subscripts on the LHS of the equations are defined for $u>0,v=0_{-}$ and are Laplace transforms in η, while the T,v quantities with upper-case subscripts are defined for $u=0_{+},v<0$ and are Laplace transforms in $-m_{pb},-m_{sb},-m_{sb}$, respectively, on the RHS of (5.18), (5.19), (5.20). It yields:

5.18 $$\begin{array}{rl} & Z_{o}[2\xi_{p}(\eta v_{x}+\alpha_{o}v_{z})-v_{y}({\alpha_{o}}^{2}+\eta^{2}-\xi_{s}^{2})]-k_{s}(T_{yy}\xi_{p}+\eta T_{xy}+\alpha_{o}T_{yz})\\ & \;\;=Z_{o}[-v_{Y2}({\alpha_{o}}^{2}+{k_{p}}^{2}-{k_{s}}^{2})+v_{X2}\sin(2\gamma )(\eta^{2}-\xi_{p}^{2})+2\xi_{p}(\eta v_{X2}\cos(2\gamma )\\ & \;\;+\eta v_{Y2}\sin(2\gamma )+\alpha_{o}v_{Z2}\cos(\gamma ))+v_{Y2}\cos(2\gamma )(\xi_{p}^{2}-\eta^{2})+2\alpha_{o}\eta v_{Z2}\sin(\gamma )]\\ & \;\;-k_{s}[\xi_{p}(T_{Y2Y2}\cos(\gamma )-T_{X2Y2}\sin(\gamma ))+\eta T_{X2Y2}\cos(\gamma )+\eta T_{Y2Y2}\sin(\gamma )+\alpha_{o}T_{Y2Z2}], \end{array}$$

5.19 $$\begin{array}{rl} & k_{s}\xi_{s}(\eta T_{xy}+\alpha_{o}T_{yz})-k_{s}T_{yy}({\alpha_{o}}^{2}+\eta^{2})\\ & \;\;+Z_{o}[\xi_{s}^{2}(-(\eta v_{x}+\alpha_{o}v_{z}))+2v_{y}({\alpha_{o}}^{2}+\eta^{2})\xi_{s}+({\alpha_{o}}^{2}+\eta^{2})(\eta v_{x}+\alpha_{o}v_{z})]\\ & \;\;=k_{s}\xi_{s}[\eta T_{X2Y2}\cos(\gamma )+\eta T_{Y2Y2}\sin(\gamma )+\alpha_{o}T_{Y2Z2}]\\ & \;\;-k_{s}({\alpha_{o}}^{2}+\eta^{2})[T_{Y2Y2}\cos(\gamma )-T_{X2Y2}\sin(\gamma )]\\ & \;\;+Z_{o}\{\xi_{s}[-\xi_{s}(\eta v_{X2}\cos(2\gamma )+\eta v_{Y2}\sin(2\gamma )+\alpha_{o}v_{Z2}\cos(\gamma ))-v_{X2}({\alpha_{o}}^{2}+2\eta^{2})\sin(2\gamma )\\ & \;\;+v_{Y2}({\alpha_{o}}^{2}+2\eta^{2})\cos(2\gamma )+{\alpha_{o}}^{2}v_{Y2}-2\sin(\gamma )\alpha_{o}\eta v_{Z2}]\\ & \;\;+({\alpha_{o}}^{2}+\eta^{2})[\eta v_{X2}\cos(2\gamma )+\eta v_{Y2}\sin(2\gamma )+\alpha_{o}v_{Z2}\cos(\gamma )]\} \end{array}$$

5.20 $$\begin{array}{rl} \text{and}\; & \;-{k_{s}}^{3}T_{yz}+\xi_{s}\{Z_{o}[{k_{s}}^{2}v_{z}-2\alpha_{o}v_{y}\xi_{s}-2\alpha_{o}(\eta v_{x}+\alpha_{o}v_{z})]+\alpha_{o}k_{s}T_{yy}\}\\ & \;\;+\alpha_{o}k_{s}(\eta T_{xy}+\alpha_{o}T_{yz})=Z_{o}\{\alpha_{o}\sin(2\gamma )[v_{X2}(-{\alpha_{o}}^{2}-2\eta^{2}+{k_{s}}^{2})-2\alpha_{o}\eta v_{Y2}\xi_{s}]\\ & \;\;+\alpha_{o}\cos(2\gamma )[v_{Y2}({\alpha_{o}}^{2}+2\eta^{2}-{k_{s}}^{2})+-2\eta v_{X2}\xi_{s}]\\ & \;\;+v_{Z2}\cos(\gamma )({k_{s}}^{2}-2{\alpha_{o}}^{2})\xi_{s}+\eta v_{Z2}\sin(\gamma )({k_{s}}^{2}-2{\alpha_{o}}^{2})+\alpha_{o}v_{Y2}({\alpha_{o}}^{2}-{k_{s}}^{2})\}\\ & \;\;+k_{s}\{T_{Y2Z2}({\alpha_{o}}^{2}-{k_{s}}^{2})+\alpha_{o}\xi_{s}[T_{Y2Y2}\cos(\gamma )-T_{X2Y2}\sin(\gamma )]\\ & \;\;+\alpha_{o}\eta [T_{X2Y2}\cos(\gamma )+T_{Y2Y2}\sin(\gamma )]\}. \end{array}$$

We remark that (5.18)–(5.20) are the spectral functional equations of region 2 for an elastic wave motion problem in an isotropic medium at skew (non-planar) incidence ($\alpha_{o}\neq 0$). As cross-validation, we note that (5.18)–(5.20) of region 2 are equivalent to (5.11)–(5.13) of region 1, according to the following replacements dictated by means of symmetry (figure 2):

5.21 $$\begin{array}{rl} & \;\{v_{x},v_{y},v_{z},T_{yy},T_{xy},T_{yz}\}\to \{v_{x},-v_{y},v_{z},T_{yy},-T_{xy},-T_{yz}\},\\ & \;\;\{v_{X2},v_{Y2},v_{Z2},T_{Y2Y2},T_{X2Y2},T_{Y2Z2}\}\to \{v_{X},-v_{Y},v_{Z},T_{YY},-T_{XY},-T_{YZ}\}. \end{array}\}$$

The procedure reported in this section can be repeated to get the functional equations for regions 3 and 4 following also the explicit mathematical steps described in [1] for em applications.

## 6. Validation of functional equations for an isotropic angular region with traction-free boundary conditions in the two-dimensional case

The functional equations for the two-dimensional (planar and anti-planar) problems ($\alpha_{o}=0$) are a particular case of the ones obtained for the general three-dimensional problem (5.11)–(5.13) and (5.18)–(5.20), respectively, for region 1 and region 2 with reference to figure 2*b*.

Taking into consideration region 1, in the following, we demonstrate that the GWHEs obtained from the proposed functional equations while enforcing the traction-free face boundary conditions in the planar angular problem ($\alpha_{o}=0$) and the functional equations obtained in [14] by Gautesen’s group are identical, although the applied notations are very different from each other and cumbersome to be compared. Moreover, the functional equation for the anti-planar problem are checked with an independent method, too.

We recall that the explicit functional equations for region 1 reported in (5.11)-(5.13) are derived from (4.16). Since functional equations can be written up to multiplicative constant as eigenvectors, to perform the comparison with compact expressions and to avoid the lack of definition of some eigenvectors/reciprocal vectors for $\alpha_{o}=0$, we redefine the reciprocal vectors (2.33) as in the following scaling:

6.1 $$\begin{array}{rl} & \nu_{1}=\frac{2\xi_{p}k_{s}^{2}V(1,:)}{\alpha_{o}},\;\nu_{2}=\frac{2\xi_{s}k_{s}^{2}V(2,:)}{\eta };\\ & \;\nu_{3}=2V(3,:),\;\nu_{4}=\frac{2\xi_{p}k_{s}^{2}V(4,:)}{\alpha_{o}},\\ & \;\nu_{5}=\frac{2\xi_{s}k_{s}^{2}V(5,:)}{\eta },\;\nu_{6}=2V(6,:). \end{array}$$

For readability, we report (6.1) in explicit form for $\alpha_{o}=0$ in terms of rows of the following matrix:

6.2 $$V_{o}=\left(\begin{array}{cccccc} -\frac{k_{s}\xi_{p}}{Z_{o}} & 0 & -\frac{\eta k_{s}}{Z_{o}} & 2\eta \xi_{p} & \xi_{s}^{2}-\eta^{2} & 0\\ -\frac{\eta k_{s}}{Z_{o}} & 0 & \frac{k_{s}\xi_{s}}{Z_{o}} & \eta^{2}-\xi_{s}^{2} & 2\eta \xi_{s} & 0\\ 0 & -\frac{k_{s}}{Z_{o}\xi_{s}} & 0 & 0 & 0 & 1\\ -\frac{k_{s}\xi_{p}}{Z_{o}} & 0 & \frac{\eta k_{s}}{Z_{o}} & 2\eta \xi_{p} & \eta^{2}-\xi_{s}^{2} & 0\\ \frac{\eta k_{s}}{Z_{o}} & 0 & \frac{k_{s}\xi_{s}}{Z_{o}} & \xi_{s}^{2}-\eta^{2} & 2\eta \xi_{s} & 0\\ 0 & \frac{k_{s}}{Z_{o}\xi_{s}} & 0 & 0 & 0 & 1 \end{array}\right).$$

For $\alpha_{o}=0,$ we obtain a simplified version of (5.6)

6.3 $$\begin{array}{rl} & \psi_{as}(v)\\ & \;\;=\left(\begin{array}{c} T_{XY}\sin(\gamma )+T_{YY}\cos(\gamma )\\ \frac{\eta v_{Z}\sin(\gamma )Z_{o}}{k_{s}}+T_{YZ}\\ \frac{4\eta Z_{o}\sin(\gamma )({k_{p}}^{2}-{k_{s}}^{2})(v_{Y}\sin(\gamma )-v_{X}\cos(\gamma ))}{{k_{s}}^{3}}+T_{XY}\cos(\gamma )-T_{YY}\sin(\gamma )\\ v_{X}\cos(2\gamma )-v_{Y}\sin(2\gamma )\\ \frac{v_{Y}({k_{p}}^{2}\cos(2\gamma )-{k_{p}}^{2}+{k_{s}}^{2})+{k_{p}}^{2}v_{X}\sin(2\gamma )}{{k_{s}}^{2}}\\ v_{Z}\cos(\gamma ) \end{array}\right). \end{array}$$

With reference to figure 1, we now obtain the explicit functional equations (4.16) of an angular region filled by isotropic elastic medium before imposing face boundary conditions in the two-dimensional case.

With $\alpha_{o}=0$, the re-scaled reciprocal vectors (6.2), the Laplace transform ${\tilde{\psi }}_{t}(\eta ,v=0)$ (4.1) of the continuous field (2.17) at face o and the Laplace transform ${\overset{⌣}{\psi }}_{as}^{\;}(\chi )$ (4.15) of the quantity (6.3) expressed in terms of the continuous field at face a, we obtain the following explicit form of the functional equations (4.16):

6.4 $$\begin{array}{rl} & \frac{k_{s}(\eta T_{xy}-T_{yy}\xi_{p})}{Z_{o}}+2\eta v_{x}\xi_{p}+v_{y}(\eta^{2}-\xi_{s}^{2})\\ & \;\;=\sin(2\gamma )[-2\eta \xi_{p}v_{Y}-v_{X}{\xi_{p}}^{2}+\eta^{2}v_{X}]+v_{Y}({k_{p}}^{2}-{k_{s}}^{2})\\ & \;\;+\cos(2\gamma )[-{\xi_{p}}^{2}v_{Y}+2\eta \xi_{p}v_{X}+\eta^{2}v_{Y}]\\ & \;\;-\frac{k_{s}\xi_{p}[T_{XY}\sin(\gamma )+T_{YY}\cos(\gamma )]+\eta k_{s}[T_{XY}\cos(\gamma )-T_{YY}\sin(\gamma )]}{Z_{o}}, \end{array}$$

6.5 $$\begin{array}{rl} & \frac{k_{s}(T_{xy}\xi_{s}+\eta T_{yy})}{Z_{o}}-v_{x}(\eta^{2}-\xi_{s}^{2})+2\eta v_{y}\xi_{s}\\ & \;\;=\sin(2\gamma )[2\eta v_{X}\xi_{s}-v_{Y}\xi_{s}^{2}+\eta^{2}v_{Y}]+\cos(2\gamma )[v_{X}\xi_{s}^{2}+2\eta v_{Y}\xi_{s}-\eta^{2}v_{X}]\\ & \;\;+\frac{k_{s}\xi_{s}[T_{XY}\cos(\gamma )-T_{YY}\sin(\gamma )]+k_{s}\eta [T_{XY}\sin(\gamma )+T_{YY}\cos(\gamma )]}{Z_{o}}, \end{array}$$

6.6 $$\begin{array}{rl} & \frac{k_{s}T_{yz}}{Z_{o}\xi_{s}}+v_{z}=\frac{k_{s}T_{YZ}}{Z_{o}\xi_{s}}+\frac{\eta v_{Z}}{\xi_{s}}\sin(\gamma )+v_{Z}\cos(\gamma ). \end{array}$$

We recall the T,v quantities with lower-case subscripts on the LHS of the equations are defined for $u>0,v=0_{+}$ and are Laplace transforms in η of ${\tilde{\psi }}_{t}(\eta ,v=0)$, while the T,v quantities with upper-case subscripts are defined for $u=0_{+}$, v>0 and are Laplace transforms in $-m_{p}$, $-m_{s}$, $-m_{s}$ of $\psi_{as}(v),$ respectively, on the RHS of (6.4), (6.5), (6.6).

We note that (6.4) is related to the complex propagation constant $-m_{p}$ of the principal wave while (6.5), (6.6) are related to $-m_{s}$, i.e. the one of the secondary waves.

We note also some sort of symmetry between (6.4) and (6.5) except for the additional term $v_{Y}({k_{p}}^{2}-{k_{s}}^{2})$ in (6.4).

Equations (6.4), (6.5) and (6.6) are functional equations for the general two-dimensional wave motion angular problem in elasticity before imposing boundary conditions, i.e. they represent the planar and anti-planar problems.

To complete the validation with the equations proposed at (4.1) of [14], with reference to region 1 of figure 1, we impose traction-free face boundary conditions at faces o and a, i.e. the traction $\text{t}=\underline{T}\cdot \text{n}=\text{0}$ where n is the unit normal to the face

6.7 $$\begin{array}{rl} & T_{yy},T_{yz},\;T_{yx}=0\;\text{at face o}\;(u>0,v=0_{+})\\ & \;T_{YY},\;T_{YZ},T_{YX}=0\;\text{at face a}\;(u=0_{+},v>0). \end{array}$$

It yields the following GWHEs:

6.8 $$\begin{array}{rl} & 2\eta v_{x}\xi_{p}+v_{y}(\eta^{2}-\xi_{s}^{2})\\ & \;\;=\sin(2\gamma )[-2\eta \xi_{p}v_{Y}+v_{X}(\eta^{2}-{\xi_{p}}^{2})]\\ & \;\;+\cos(2\gamma )[v_{Y}(\eta^{2}-{\xi_{p}}^{2})+2\eta \xi_{p}v_{X}]+v_{Y}({k_{p}}^{2}-{k_{s}}^{2}), \end{array}$$

6.9 $$\begin{array}{rl} & \;-v_{x}(\eta^{2}-\xi_{s}^{2})+2\eta v_{y}\xi_{s}=\sin(2\gamma )[2\eta v_{X}\xi_{s}-v_{Y}\xi_{s}^{2}+\eta^{2}v_{Y}]\\ & \;\;+\cos(2\gamma )[v_{X}\xi_{s}^{2}+2\eta v_{Y}\xi_{s}-\eta^{2}v_{X}] \end{array}$$

6.10 $$\begin{array}{rl} \text{and}\; & v_{z}=\frac{\eta v_{Z}}{\xi_{s}}\sin(\gamma )+v_{Z}\cos(\gamma ). \end{array}$$

where the v quantities with lower-case subscripts on the LHS of (6.8), (6.9) and (6.10) are plus functions in η and v quantities with upper-case subscripts on the RHS are minus functions (plus functions) in $m_{p},m_{s},m_{s}$ ($-m_{p},-m_{s},-m_{s}$). Both minus and plus functions are Laplace transforms. Standard plus(minus) functions are analytic in the upper(lower) half-plane. We extend the theory to non-standard functions when they have isolated poles due to plane wave sources located in the standard regularity half-plane.

Note that (6.10) is independent from (6.8), (6.9). In fact, (6.10) is associated with the SH wave in the wave motion problem (anti-planar problem), while (6.8), (6.9) model the coupled problem between P and SV waves (planar problem).

Equation (6.10) can be checked and validated after imposing the traction-free face boundary conditions with (3.15.5) of [4], where a completely different method specialized on anti-planar problems has been used. Now, let us compare (6.8), (6.9) with (4.1) of [14], reported in original form at (6.11) with (6.12)–(6.13).

6.11 $$\begin{array}{rl} & \begin{array}{rl} & a(\xi ){\hat{u}}_{1}(\xi )-b_{1}(\xi ){\hat{u}}_{2}(\xi )+{\hat{U}}_{1}(\xi )=f_{1}(\xi )\\ & b_{2}(\xi ){\hat{u}}_{1}(\xi )+a(\xi ){\hat{u}}_{2}(\xi )+{\hat{U}}_{2}(\xi )=f_{2}(\xi ), \end{array}\} \end{array}$$

6.12 $$\begin{array}{rl} & \begin{array}{rl} & {\hat{U}}_{1}(\xi )={\left(-1\right)}^{\ell }[-a(\zeta_{1}){\hat{u}}_{1}(\zeta_{1})+{\bar{b}}_{1}(\xi ){\hat{u}}_{2}(\zeta_{1})],\;\ell =1,2,\;\text{(antisym, sym) }\\ & {\hat{U}}_{2}(\xi )={\left(-1\right)}^{\ell }[{\bar{b}}_{2}(\xi ){\hat{u}}_{1}(\zeta_{2})+a(\zeta_{2}){\hat{u}}_{2}(\zeta_{2})],\;\ell =1,2\;\text{(antisym, sym) } \end{array}\} \end{array}$$

6.13 and ζ1,2=ξcos⁡α+γ1,2(ξ)sin⁡α, η1,2=ξsin⁡α−γ1,2(ξ)cos⁡α b¯1,2(ξ)=2ζ1,2η1,2.}

In (6.11), ${\hat{u}}_{1}(\xi )$, ${\hat{u}}_{2}(\xi )$ are one-sided Fourier transforms of unknown displacements on face o (figure 1), respectively, in x,y, ξ is the spectral variable, a(ξ), $b_{1}(\xi )$, $b_{2}(\xi )$ are spectral functions and ${\hat{U}}_{1}(\xi )$, ${\hat{U}}_{2}(\xi )$ are one-sided Fourier transforms of quantities defined in terms of unknown displacements on face a (figure 1), respectively, in X, −Y. $f_{1}(\xi )$, $f_{2}(\xi )$ model the source of the wave motion problem. In order to compare (6.11) with (6.8), (6.9), we scale all the displacements by jω to get the velocities, thus (6.11) hold in homogeneous form ($f_{1}(\xi ),f_{2}(\xi )=0$) also interpreting ${\hat{u}}_{i}(\xi )$, ${\hat{U}}_{i}(\xi )$ in terms of velocities. Moreover, we observe that i=1,2 waves in [14] are respectively associated with SV,P waves, thus we need to compare (6.8), (6.9), respectively, with the 2nd and the first equation of (6.11). With the help of the definitions given in [14], let us interpret (6.11) in our formalism. Table 1 reports the correspondences for the definition of some quantities in the two works. With table 1, it is easy to show the equivalence between the LHS of (6.8), (6.9) and the terms in ${\hat{u}}_{i}(\xi )$ in (6.11).

**Table 1.** Translation of definitions between this work and [14]

[14]	ξ	$\kappa_{1,2}$	α	${\hat{u}}_{1,2}(\xi )$	$\gamma_{1,2}^{2}=\kappa_{1,2}^{2}-\xi^{2}$	$a(\xi )=\kappa_{1}^{2}-2\xi^{2}$	$b_{1,2}(\xi )=2\xi \gamma_{1,2}(\xi )$
this paper	η	$k_{s,p}$	γ	$v_{x,y}(\eta )$	$\xi_{s,p}^{2}=k_{s,p}^{2}-\eta^{2}$	$\xi_{s}^{2}-\eta^{2}$	$2\eta \xi_{s,p}$

To complete the comparison we need to check the first equation of (6.11) and (6.9) focusing attention on ${\hat{U}}_{1}(\xi )$ (6.12) and then check the second equation of (6.11) and (6.8) focusing attention on ${\hat{U}}_{2}(\xi )$ (6.12). Starting from (6.13), $\zeta_{1,2}$ play the roles of $-m_{s,p}$ (4.17) and $\eta_{1,2}$ play the role of $n_{s,p}$. In particular, we note that, in our notation,

6.14 $$\zeta_{1,2}\to \eta \cos\gamma +\xi_{s,p}\sin\gamma \;\text{and}\;\eta_{1,2}\to \eta \sin\gamma -\xi_{s,p}\cos\gamma ,$$

that apart from a sign in the combination of the two terms are, respectively, $-m_{s,p}$ (4.17) and $n_{s,p}$:

6.15 $$m_{s,p}=-\eta \cos\gamma +\xi_{s,p}\sin\gamma \;\text{and}\;n_{s,p}=\eta \sin\gamma +\xi_{s,p}\cos\gamma .$$

Further sign differences appear also in the combination of the quantities between (6.8)–(6.9) and (6.11). We are convinced that these differences are due to different notations in Fourier transforms between engineering (ours, [7] p. XV) and applied mathematics (as in [14]) and, to the different orientation of local coordinate system on face a between our work and [14] where (X,−Y) are selected (figure 1). We note that ${\hat{u}}_{1,2}(\zeta_{1})$ in ${\hat{U}}_{1}(\xi )$ (6.12) for equation (6.11) play the roles of $v_{X,Y}(-m_{s})$ for equation (6.9). Let us compare the functional coefficient of ${\hat{u}}_{1,2}(\zeta_{1})$ with the ones of $v_{X,Y}(-m_{s})$. With the help of table 1 and (6.14)–(6.15), for ${\hat{u}}_{1}(\zeta_{1})$ and $v_{X}(-m_{s})$, we have respectively,

6.16 $$-a(\zeta_{1})=\kappa_{1}^{2}-2\zeta_{1}^{2}\to k_{s}^{2}-2m_{s}^{2}$$

and

6.17 $$\sin(2\gamma )2\eta \xi_{s}+\cos(2\gamma )[\xi_{s}^{2}-\eta^{2}]=k_{s}^{2}-2m_{s}^{2}$$

after some trigonometric manipulation. Again for ${\hat{u}}_{2}(\zeta_{1})$ and $v_{Y}(-m_{s}),$ we have, respectively,

6.18 $${\bar{b}}_{1}(\xi )=2\zeta_{1}\eta_{1}\to 2m_{s}n_{s}$$

and

6.19 $$\sin(2\gamma )[-\xi_{s}^{2}+\eta^{2}]+\cos(2\gamma )[2\eta \xi_{s}]=2m_{s}n_{s}.$$

Now let us complete the comparison between the second equation of (6.11) and (6.8), focusing the attention on ${\hat{U}}_{2}(\xi )$ (6.12) and comparing the functional coefficient of ${\hat{u}}_{1,2}(\zeta_{1})$ in ${\hat{U}}_{2}(\xi )$ with the ones of $v_{X,Y}(-m_{p})$. With the help of table 1 and (6.14)–(6.15), for ${\hat{u}}_{1}(\zeta_{2})$ and $v_{X}(-m_{p})$, we have, respectively

6.20 $${\bar{b}}_{2}(\xi )=2\zeta_{2}\eta_{2}\to 2m_{p}n_{p}$$

and

6.21 $$\sin(2\gamma )[-{\xi_{p}}^{2}+\eta^{2}]+\cos(2\gamma )[2\eta \xi_{p}]=2m_{p}n_{p}$$

with the same calculus as done in (6.18)–(6.19). On the contrary, we note that ${\hat{u}}_{2}(\zeta_{2})$ and $v_{Y}(-m_{p})$ show different properties with respect to (6.16)–(6.17). Their respective functional coefficients are

6.22 $$a(\zeta_{2})=\kappa_{1}^{2}-2\zeta_{2}^{2}\to k_{s}^{2}-2m_{p}^{2}$$

and

6.23 $$\sin(2\gamma )[-2\eta \xi_{p}]+\cos(2\gamma )[-{\xi_{p}}^{2}+\eta^{2}]+({k_{p}}^{2}-{k_{s}}^{2})=k_{s}^{2}-2m_{p}^{2}$$

that are equivalent after some trigonometric manipulation. Note in (6.22)–(6.23), we have the simultaneous presence of SV and P spectral variables and propagation constants, and the presence of additional term $\left({k_{p}}^{2}-{k_{s}}^{2}\right)$ on the LHS of (6.23) with respect to the LHS of (6.17). This property denotes coupling between SV and P waves.

We conclude by affirming that (6.8), (6.9), (6.10) are the GWHEs for the elastic wave motion angular problem in two dimensions ($\alpha_{o}=0$) with traction-free face boundary conditions that model the planar (6.8), (6.9) and anti-planar (6.10) problems in the presence of plane-wave sources or sources located at infinity with the help of the concept of non-standard Laplace transforms (see §1.4 of [5]).

See the electronic supplementary material for a report of the validation of functional equations by evaluating the characteristic impedances of half spaces in planar problems.

## 7. Remarks and conclusion

In this work, we have introduced a general method for the deduction of spectral functional equations and thus GWHEs in angular regions filled by arbitrary linear isotropic homogeneous media in elasticity. The importance to formulate wedge problems with GWHEs in electromagnetism has been shown in [4,5]. We remark that these equations are important also for elastic wedge problems. In particular, the functional equations obtained and solved in [14] by Gautesen’s group for the planar elastic wedge are GWHEs, although not defined in this way.

The method is based on the original solution of vector differential equations of first order via dyadic Green’s function method and on the projection of this solution along the boundaries of the angular region using reciprocal vectors of the pertinent algebraic matrix related to the matrix differential operator. The application of the boundary conditions to the functional equations yields GWHEs for practical problems. We observe that the functional equations are the starting point to develop solutions using the WH technique for complex scattering problems.

Using the concept of non-standard Laplace transforms (see §1.4 of [5]), the validity of the functional equations and of the GWHEs obtained in the absence of sources is extended to the total fields in the presence of plane-wave sources or in general of sources located at infinity. We observe that the GWHEs in elasticity contain unknowns defined in multiple complex planes $\eta ,-m_{p},-m_{s}$ related to P and S waves and this property recalls electromagnetic applications (and related solution methods) in media with multiple propagation constants as reported in [25–28]. In fact, in this case, the reduction of GWHEs to classical WH equations is not possible. Explicit expressions of spectral functional equations in algebraic form are provided in the text in the general case of non-planar elastic problems in angular regions with isotropic media and arbitrary boundary conditions and, we remark that, according to our opinion, this is the first time in the literature. Validation of the GWHE formulation has been demonstrated by comparison with prestigious literature references reporting special simplified cases in anti-planar and planar problems. The paper demonstrates the flexibility and the advantages of the proposed method, based on first-order differential formulation, that is useful for the analysis of complex scattering problems containing angular regions in arbitrarily linear media by changing the matrix operator defined through the fundamental matrices $M_{o}$, $M_{1}$, $M_{2}$. The paper shows systematic procedural steps that can be used for arbitrary wave motion problems in different physics.

## Footnotes

1 The GWHEs differ from the Classical Wiener–Hopf equations (CWHEs) for the definitions of the unknowns in spectral domain. While CWHEs introduce plus and minus functions that are always defined in the same complex plane, the GWHEs present plus and minus functions that are defined in different complex planes but related together.

2 A square matrix of dimension *n* is called semisimple iff it has a basis of eigenvectors in $R^{n}$.

3 Any dyadic $\underline{A}=\sum_{ij}A_{ij}{\text{e}}_{\text{i}}{\text{e}}_{\text{j}}$ can be represented by a matrix A with elements $A_{ij}$ where ${\text{e}}_{\text{i}}$ are unit vectors and vice versa.

## Data accessibility

This article has no additional data.

## Glossary

**Table 2.** Symbols introduced in the paper

notation	description
(x,y,z), (r,φ,z), (u,v,z), (X,Y,Z)	Cartesian, cylindrical, oblique Cartesian, local to face Cartesian coordinates
A, A, $\underline{A}$, A, A(⋅,⋅)	scalar, column vector, dyadic, matrix, matrix differential operator
$k_{p},k_{s}$	propagation constants of P and S waves
$\underline{T}\;(\text{T})$, $\underline{S}\;(\text{S})$	stress tensor (Voigt notation), strain tensor (Voigt notation)
p, v	vector momentum density, vector particle velocity
ρ, λ, μ	material density and Lame’s constants
$\underline{\underline{C}}$	Hooke’s Law as fourth-order stiffness tensor
$\nabla_{T}$, $\nabla_{v}$, $\Gamma_{\nabla }$	matrix differential operators
ψ, θ	vector fields in abstract notation
W	matrix constitutive parameters of media
$\psi_{t}$	transverse field for a stratification along the *y*-direction
$M(\frac{\partial }{\partial z},\frac{\partial }{\partial x})$	transversal matrix differential operator for elastic equations
$D_{x}=\frac{\partial }{\partial x}$	alternative partial derivative notation
$\alpha_{o}$	field dependence specified by the factor $e^{-j\alpha_{o}\;z}$ due to invariance along z
η	Fourier or Laplace spectral variable according to the position on the text
$\Psi_{t}(\eta )$	Fourier transform along x=u direction (y,z or v,z dependence is omitted)
M(η)	matrix operator in Fourier/Laplace domain in indefinite rectangular region
$\lambda_{i},\;{\text{u}}_{i},\;\nu_{i}$	eigenvalues, eigenvector and reciprocal vectors of M(η)
$\xi_{i}$	different notation of $\lambda_{i}$ for propagation’s properties, multivalued function
γ	aperture angle of angular regions (figure 1)
$M_{e}(\gamma ,\eta )$	matrix operator in Fourier/Laplace domain in indefinite angular region
$\lambda_{ei}$	eigenvalues of $M_{e}(\gamma ,\eta )$
${\tilde{\psi }}_{t}(\eta ,v)$	Laplace transform along x≡u of $\psi_{t}(u,v)$ (omitting z dependence)
$\psi_{s}(v)$	field components on the face of an angular region in Laplace domain
$\psi_{as}(v)$, ${\overset{⌣}{\psi }}_{as}^{\;}(\chi )$	specialized expression of $\psi_{s}(v)$ on face a and its Laplace transform
$\underline{G}(v,v^{\prime })$	dyadic Green’s function in Laplace domain for an angular region
$m_{ai}$	spectral variable for the evaluation of ${\overset{⌣}{\psi }}_{as}^{\;}(\chi )$ along face a in functional eqs
